# Interfacial magnetic reconstruction and emergent metallicity in oxide heterostructures

**DOI:** 10.1038/s43246-026-01275-x

**Published:** 2026-07-14

**Authors:** Zengxing Lu, Jiatai Feng, Xuan Zheng, You-Guo Shi, Run-Wei Li, Nicholas C. Plumb, Carmine Autieri, Mario Cuoco, Zhiming Wang, Milan Radovic

**Affiliations:** 1 Ningbo Institute of Materials Technology and Engineering, Chinese Academy of Sciences, Ningbo, China; 2 Materials Genome Institute, Shanghai University, Shanghai, China; 3 Center for Transformative Science, ShanghaiTech University, Shanghai, China; 4 Institute of Physics, Chinese Academy of Sciences, Beijing, China; 5 School of Materials Science and Engineering, Eastern Institute of Technology, Ningbo, China; 6 PSI Center for Photon Science, Paul Scherrer Institute, Villigen, Switzerland; 7 International Research Centre Magtop, Institute of Physics, Polish Academy of Sciences, Warsaw, Poland; 8 CNR-SPIN, UOS Salerno, Salerno, Fisciano Italy; 9 Center of Materials Science and Optoelectronics Engineering, University of Chinese Academy of Sciences, Beijing, China

**Keywords:** Condensed-matter physics, Materials science

## Abstract

Interfaces between correlated oxides and strong spin–orbit coupled materials provide a fertile platform for emergent quantum phases, yet controlling these phases through interfacial magnetic reconstruction remains a major challenge. Here, we observe emergent metallicity in ultrathin SrIrO_3_/SrRuO_3_ heterostructures formed from insulating constituent layers, and this transition is closely associated with interfacial magnetic reconstruction. Combining transport, magnetometry, and angle-resolved photoemission spectroscopy, supported by first-principles calculations, we show that finite spectral weight at the Fermi level and ferromagnetic signatures emerge in the heterostructures. Our calculations further suggest that a staggered Dzyaloshinskii–Moriya interaction at the interface favors ferromagnetic order in SrRuO₃ and collinear antiferromagnetism in SrIrO₃, relative to the collinear and noncollinear antiferromagnetism in isolated ultrathin SrRuO₃ and SrIrO₃, respectively, providing a possible microscopic framework for the coupled magnetic and electronic reconstruction. Our findings identify interfacial magnetic interactions as a powerful tuning parameter for controlling correlated electronic phases in spin-orbit-coupled oxides, opening pathways toward the design of emergent quantum and topological states.

## Abstract (web summary)

Interfaces between correlated oxides and spin–orbit-coupled materials hold promise for novel quantum phases, yet controlling these phases remains challenging. Here, the authors demonstrate emergent metallicity in SrIrO₃/SrRuO₃ heterostructures, linked to interfacial magnetic reconstruction, highlighting interfacial magnetic interactions as a key tool for tuning electronic phases in quantum materials.

## Introduction

Transition-metal oxides host an exceptionally rich spectrum of magnetic ground states, ranging from ferromagnetism (FM) and antiferromagnetism (AFM) to the recently identified altermagnetic (AM) phase. These magnetic phases are intimately intertwined with the underlying electronic structure, giving rise to insulating, semiconducting, and metallic behaviors within the same material family. The strong coupling among charge, spin, orbital, and lattice degrees of freedom enables wide tunability yet also means that small perturbations can drive qualitatively distinct electronic and magnetic states. Understanding how to stabilize, manipulate, or combine these magnetic symmetries is therefore a central challenge in modern condensed matter physics.

Among the diverse magnetic symmetries found in transition‑metal oxides, AFM and FM exhibit particularly distinct correlations with electronic transport. In many oxides, AFM order arises from Coulomb-driven Heisenberg superexchange and is typically associated with insulating behavior, whereas insulating ferromagnetism is relatively rare because it requires a finely balanced interplay of orbital and exchange interactions^1^. Mechanisms that stabilize ferromagnetism, such as double exchange^2^ or Ruderman-Kittel-Kasuya-Yosida (RKKY) coupling^3–5^, are more effective in itinerant systems and are typically associated with metallic behavior. This dichotomy highlights a deep link between magnetic exchange and electronic itinerancy, suggesting that modifying magnetic interactions can, in principle, transform an insulator into a metal, or vice versa.

Materials with strong spin–orbit coupling (SOC) and electron correlations provide a natural platform for magnetic-reconstruction–driven insulator–metal transitions^6–9^. Among them, the perovskite oxides SrIrO_3_ (SIO) and SrRuO_3_ (SRO) are particularly notable. Although both materials are metallic in bulk, their ultrathin-film limits host antiferromagnetic insulating phases^10–17^: SrIrO₃ (SIO) exhibits a well-established noncollinear antiferromagnetic (nc-AFM) insulating state^13,18,19^, while SrRuO₃ (SRO) is likely to undergo a transition to a collinear antiferromagnetic (c-AFM) insulating state under strong dimensional confinement^20–26^. The antiferromagnetic order in both materials makes their heterostructures ideal for exploring the interplay of distinct AFM orders in proximity. Indeed, we show that ultrathin insulating SIO/SRO bilayers undergo an interfacial magnetic reconstruction that stabilizes a metallic ground state with coexisting AFM and FM orders.

To uncover the microscopic origin of this emergent phase, we combine transport measurements, magnetometry, and in-situ angle-resolved photoemission spectroscopy, supported by first-principles calculations. We find that metallic spectral weight appears at the Fermi level only when ferromagnetism is induced through a staggered Dzyaloshinskii–Moriya interaction (DMI) localized at the SIO/SRO interface. This interfacial DMI drives an AFM-to-FM reconstruction of the SRO layer and a noncollinear-to-collinear AFM reorientation in SIO. The resulting interface-engineered coexistence of distinct magnetic orders within a metallic environment provides a platform where competing magnetic symmetries can hybridize, enabling magnetic states that are otherwise inaccessible in the bulk. Our results establish interfacial magnetic interactions as a powerful and previously underexplored tuning parameter for controlling correlated electronic phases in spin-orbit-coupled oxides, opening new pathways for the design of emergent quantum and topological magnetic states.

## Results

### Engineering ferromagnetic metallicity in SIO/SRO heterostructures via interfacial DMI

**Fig. 1:**
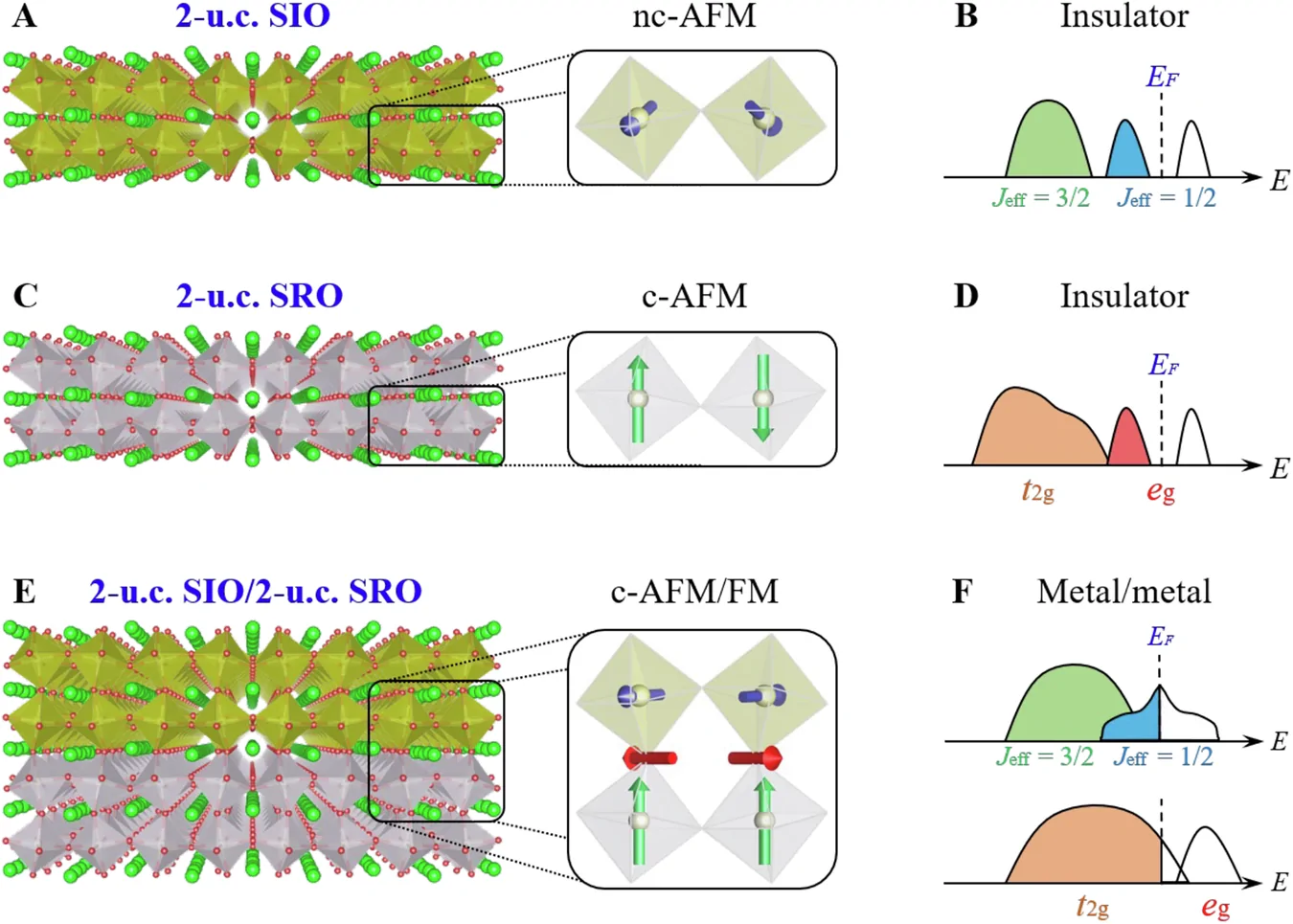
Schematic mechanism of DMI-induced insulator-to-metal transition in SIO/SRO heterostructures. Magnetic structures and density of states (DOS) of (**A**, **B**) 2-u.c. SIO, (**C**, **D**) 2-u.c. SRO, and (**E** and **F**) SIO/SRO heterostructures. Arrows represent spin moments, with blue and green colors denoting Ir and Ru moments, respectively. Ultrathin SIO and SRO are noncollinear antiferromagnetic (nc-AFM) and collinear antiferromagnetic (c-AFM) insulators, respectively, as indicated by gaps in the DOS. In SIO/SRO heterostructures, staggered DMI on apical oxygens (red arrows with opposite directions) induces magnetic reconstruction, leading to a collinear antiferromagnetic (c-AFM) state in the SIO layer and a FM state in the SRO layer. Emergent metallicity in the heterostructure, evidenced by finite DOS at the Fermi level, is a direct consequence of DMI-driven magnetic reconstruction at the SIO/SRO interface. Orbital contributions to the DOS are indicated.

**Fig. 2:**
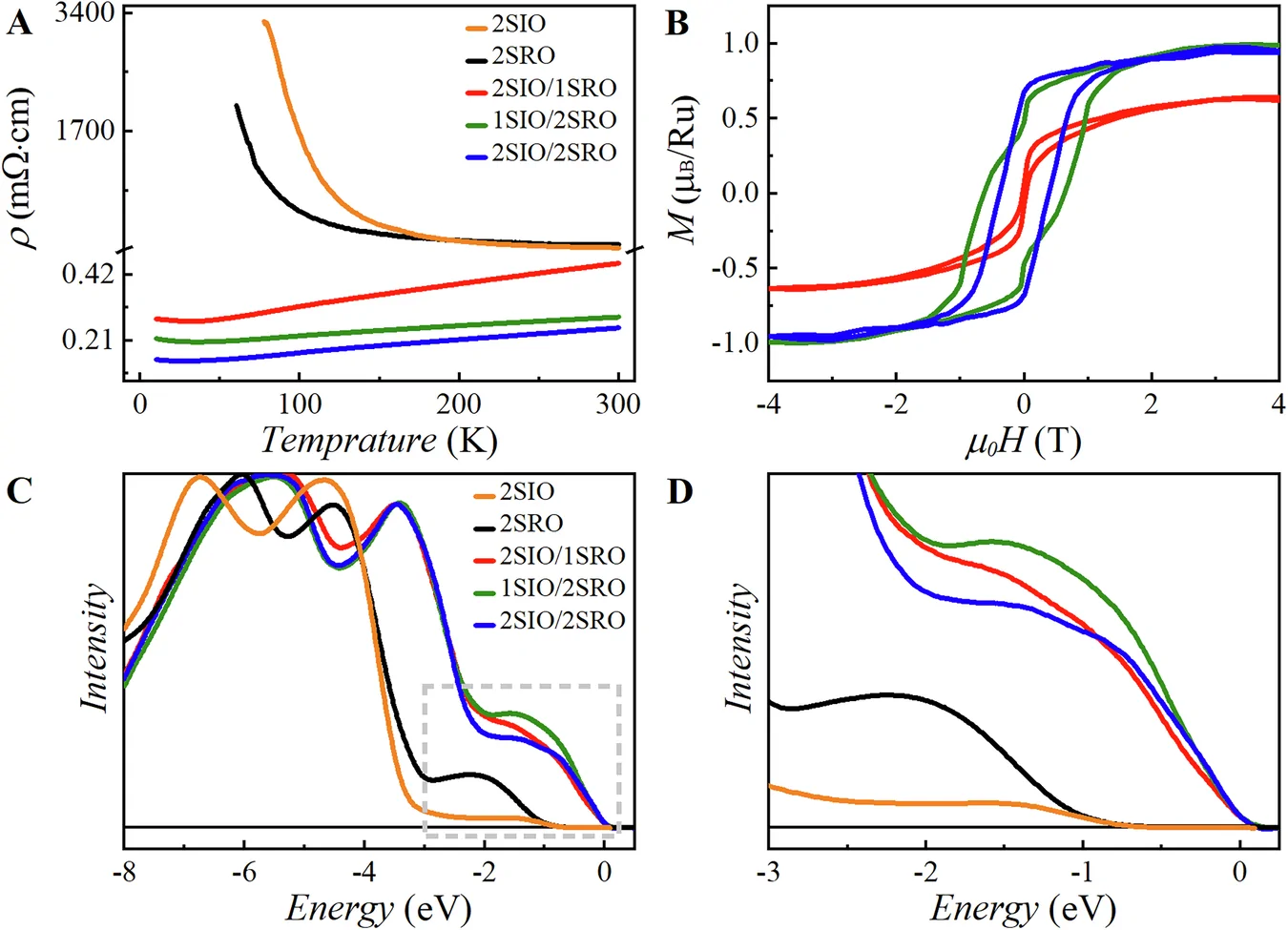
Electrical and magnetic properties of SIO/SRO heterostructures. **A** Temperature-dependent electric resistivity *ρ(T)* and (**B**) magnetization hysteresis loops *M(µ*_*0*_*H)*. **C** Valence band spectra of ultrathin bilayers compared with 2-u.c. SRO and 2-u.c. SIO monolayers. **D** Magnified plots of spectra curves in (**C**) near the Fermi level.

To understand the mechanism of magnetic reconstruction at the SIO/SRO interface, we first investigate individual SIO and SRO films, which show insulator behavior. Using density functional theory (DFT) calculations and a model Hamiltonian approach, we explore the ground state properties of ultrathin SIO and SRO films for a range of electron correlation strengths (*U*_*Ir*_ and *U*_*Ru*_). Our analysis is supported by experimental findings (Fig. 2A), indicating that below a critical thickness of 3-unit cells (u.c.), both SRO and SIO films are insulating. At the same time, isolated ultrathin SIO and SRO show distinct spin orderings (see Supplementary Information Section Ⅱ to Ⅳ for details). Specifically, the SIO favors a noncollinear antiferromagnetic (nc-AFM) state, whereas the SRO displays a collinear AFM (c-AFM) arrangement, as shown in Fig. 1A, C. These distinctions primarily arise from differences in SOC, magneto-crystalline anisotropy, and octahedral distortions inherent to each material.

The perovskite ABO_3_ structure, with space group 62, accommodates a staggered DMI associated with to octahedral tilting and rotation^22^. In bulk perovskites, where mirror symmetry is maintained, magnetic ordering is predominantly collinear. However, in SIO/SRO heterostructures, the staggered DMI (Fig. 1E) becomes different at the interface due to the broken inversion and horizontal mirror symmetries. This interfacial DMI favors specific spin configurations with magnetic moments oriented along the out-of-plane and in-plane directions. The DMI is characterized by a vector $\vec{D}$ lying along a symmetry axis within the interface plane^27,28^, and it manifests as an antisymmetric magnetic coupling between spin moments $\vec{S}$ at Ru and Ir sites, described by $\vec{D}\cdot \left({\vec{s}}_{Ru}\times {\vec{s}}_{Ir}\right)$. This interaction induces a specific spin chirality in the plane perpendicular to $\vec{D}$, which can be either left- or right-handed depending on the staggered DMI. Consequently, the DMI plays a dual role in dictating the spin ordering at the interface. First, it converts the noncollinear spin arrangement in SIO into a collinear configuration. Second, and more importantly, it drives an AFM-to-FM transition in the SRO layer. This transition arises directly from the alternating DMI vectors, which follow the staggered octahedral distortions, together with the broken inversion symmetry at the interface. The emergence of metallicity in the SIO/SRO heterostructure, evidenced by a finite density of states at the Fermi level (Fig. 1F), is therefore a direct consequence of this DMI-associated magnetic reconstruction.

### Electrical and magnetic properties of SIO/SRO heterostructures

To explore the predicted behavior stemming from calculated interfacial coupling, we investigated experimentally the electrical transport and magnetic properties of SIO/SRO heterostructures. Figure 2A shows the temperature-dependent electrical resistivity *ρ(T)* of individual 2-u.c. SIO and 2-u.c. SRO ultrathin films, alongside three representative heterostructures: 2-u.c. SIO/1-u.c. SRO, 1-u.c. SIO/2-u.c. SRO, and 2-u.c. SIO/2-u.c. SRO (hereafter denoted as 2SIO/1SRO, 1SIO/2SRO, and 2SIO/2SRO, respectively). These measurements were performed across a temperature range from 10 K to 300 K. While individual ultrathin films exhibit insulating behavior, all heterostructures demonstrate metallic temperature dependence, with conductivity increasing as the SRO layer thickness increases. This experimental observation is consistent with our theoretical calculations (Supplementary Information Fig. S6). Notably, kinks in the *ρ(T)* curves are observed at temperatures ranging from 102 K and 123 K for the heterostructures, signifying a change in electron scattering rate associated with the paramagnetic-to-ferromagnetic phase transition of the SRO layer^29,30^.

To probe the magnetic properties of the heterostructures, we performed magnetization versus out-of-plane magnetic field (*M-H*) hysteresis loop measurements (Fig. 2B). All heterostructures show clear ferromagnetic hysteresis loops, confirming the emergence of ferromagnetic order. The saturated magnetic moments are estimated to be 0.6 *µ*_B_/Ru for 2SIO/1SRO and 1 *µ*_B_/Ru for both 1SIO/2SRO and 2SIO/2SRO heterostructures. The Curie temperatures of the heterostructures, determined from temperature-dependent magnetization *M(T)* measurements (Supplementary Information Fig. S3A), are consistent with the transport measurements. By contrast, for the isolated ultrathin SIO and SRO, magnetometry does not show clear ferromagnetic hysteresis, consistent with their insulating behavior and with the absence of detectable ferromagnetic order. These results collectively demonstrate the emergence of ferromagnetic ordering and metallic behavior in the SIO/SRO heterostructures, in contrast to the insulating antiferromagnetic nature of the individual ultrathin films inferred from the first-principles calculations. Furthermore, the pronounced squareness of the out-of-plane *M-H* loops indicates that the heterostructures show perpendicular magnetic anisotropy (PMA). This conclusion is further corroborated by the well-defined anomalous Hall effect (AHE) hysteresis loops and the larger out-of-plane magnetoresistance (MR) relative to the in-plane counterpart, as shown in Supplementary Information Fig. S3B and C.

### Probing electronic structure of engineered heterostructures

To gain a comprehensive understanding of the electronic structure and the emergence of metallic states in SIO/SRO heterostructures, we performed in-situ ARPES measurements. The valence band photoemission spectra (Fig. 2C) of individual 2-u.c. SIO and 2-u.c. SRO ultrathin films reveal a suppressed spectral weight near the Fermi level and the presence of an energy gap, consistent with their insulating nature. In contrast, the SIO/SRO heterostructures exhibit a finite DOS at the Fermi level (Fig. 2D), confirming their metallic character. This observation provides direct evidence for the electronic structure reconstruction induced by the heterogeneous integration of ultrathin SIO and SRO layers.

**Fig. 3:**
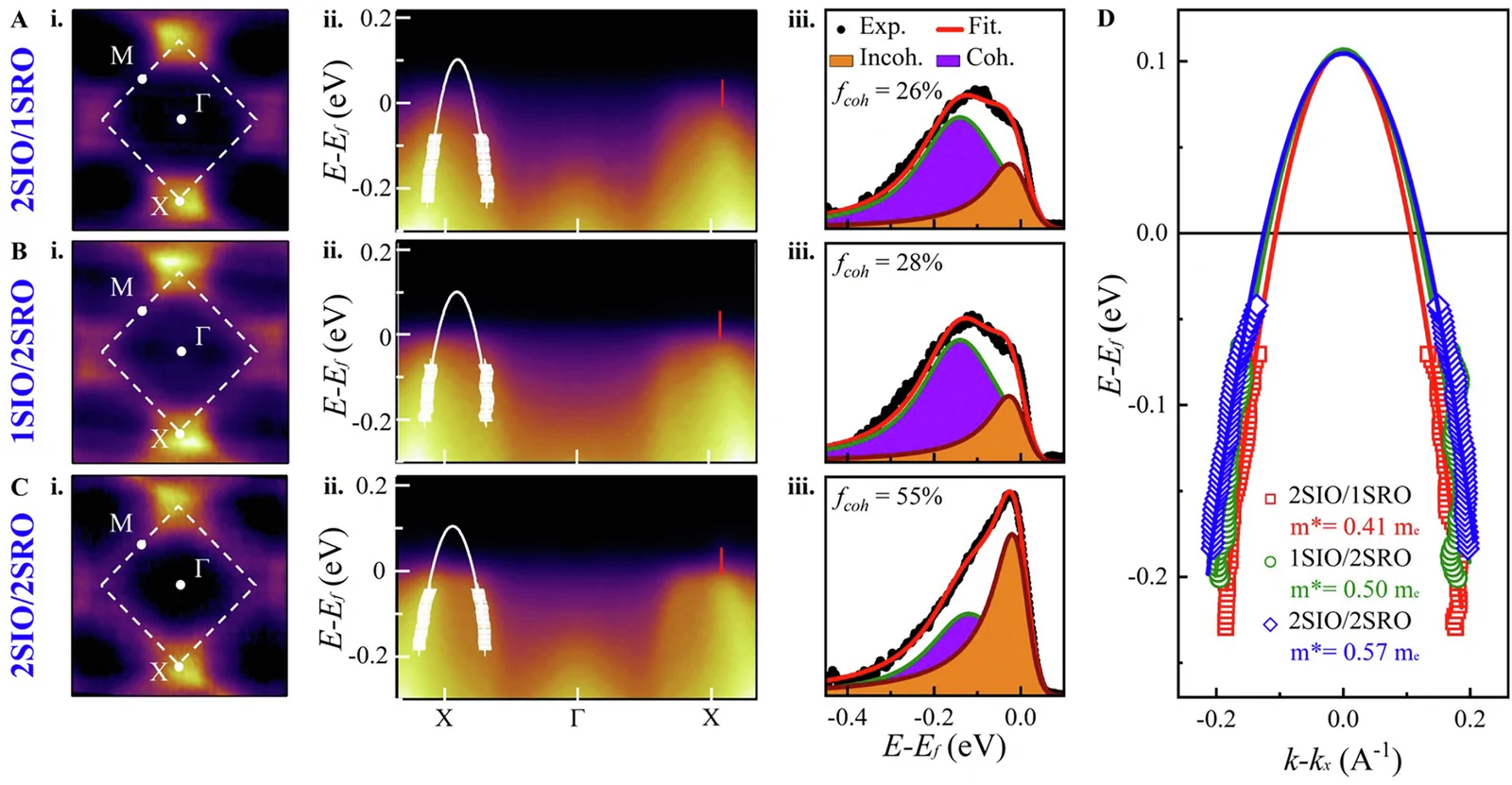
Electronic structure of SIO/SRO heterostructures probed by ARPES. Fermi surface, dispersion along X-Γ-X (white dots and plots indicate locations of EDCs), and coherent/incoherent peaks derived from EDCs around X points (marked by short red lines in dispersions) for (**A**) 1SIO/2SRO, (**B**) 2SIO/1SRO, and (**C**) 2SIO/2SRO. Exp., Fit, Incoh., and Coh. denote experiment, fit, incoherent, and coherent parts of the data, respectively. The coherent spectral weight fraction *f*_*coh*_ is calculated via ${f}_{{{\rm{coh}}}}=\frac{{W}_{{{\rm{coh}}}}}{{W}_{{{\rm{coh}}}}+{W}_{{{\rm{incoh}}}}}\times 100 \%$, using the integrated coherent and incoherent components obtained from the fitted X-point EDCs. **D** Extracted dispersion along X − Γ for films, with parabolic fits. Electronic dispersion is plotted relative to band maximum. m^∗^ and m_e_ denote electron effective mass and bare electron mass, respectively.

To further elucidate the emergent metallic states, we measured the Fermi surfaces and band dispersions of the heterostructures using ARPES with right-circularly polarized synchrotron radiation (hν = 82 eV), as shown in Fig. 3. Circular polarization enables electronic states with different orbital symmetries to be probed simultaneously within a single measurement, providing a more comprehensive view of the low-energy electronic structure of these ultrathin in-situ-grown films. The Fermi surface maps (Fig. 3 (A.i to C.i)) reveal that the electronic band crosses the Fermi level and form a well-defined Fermi surface in all three heterostructures, with a hole-like pocket centered at the X point of the Brillouin zone. The band dispersion along the high-symmetry X-Γ-X direction (Fig. 3 (A.ii to C.ii)) exhibits a clear quasiparticle peak at the Fermi level in the extracted energy distribution curves (EDCs) at the X point (Fig. 3 (A.iii to C.iii)), which is a hallmark of metallic behavior. To quantify the evolution of low-energy coherence, the X-point EDCs were fitted using the same coherent-plus-incoherent component procedure for all three heterostructures. We define the coherent spectral weight fraction as ${f}_{{{\rm{coh}}}}=\frac{{W}_{{{\rm{coh}}}}}{{W}_{{{\rm{coh}}}}+{W}_{{{\rm{incoh}}}}}\times 100$, where *W*_*coh*_ and *W*_*incoh*_ are the integrated weight of the fitted coherent and incoherent components, respectively. The *f*_*coh*_ rises from 26% (2SIO/1SRO) to 28% (1SIO/2SRO) and 55% (2SIO/2SRO), indicating an enhancement of the metallic character with increasing SRO thickness, consistent with the electrical transport results.

To quantitatively analyze the band dispersions, we extracted the momentum distribution curves (MDCs) and fitted them with a parabolic function (Fig. 3D). The effective electron mass (*m*^∗^) decreases from 0.57 m_e_ for the 2SIO/2SRO heterostructure to 0.5 m_e_ and 0.41 m_e_ for the 1SIO/2SRO and 2SIO/1SRO heterostructures, respectively, further confirming the enhanced metallicity with increased total thickness. These results demonstrate that the electronic structure of the SIO/SRO heterostructures can be effectively tuned by controlling the relative thicknesses of the SIO and SRO layers. The direct observation of a finite DOS at the Fermi level, the presence of a well-defined Fermi surface, and the evolution of effective electron mass with heterostructure thickness provide compelling evidence for the interfacial coupling-driven electronic reconstruction in these engineered oxide heterostructures.

### DMI-driven magnetic reconstruction and its impact on electronic structure

To understand the IMT undelaying mechanism in SIO/SRO heterostructures, we performed first-principles simulations based on local spin density approximations and constructed an effective model Hamiltonian. This model incorporates nearest-neighbor magnetic exchange between Ru and Ir spin moments at the SIO/SRO interface (Supplementary Information Sections Ⅱ to Ⅳ). Our analysis reveals that the staggered DMI at the interface plays a crucial role in the magnetic-reconstruction process. Specifically, in our unit cell, which contains two spins per atomic species, we focus on the DMI between Ir and Ru spins, as depicted in Fig. 1E. DMI tends to produce magnetic configurations where Ru and Ir spins are rotated by 90° with respect to each other. The simplest spin model with a staggered DMI as the dominant interaction between two layers predicts one layer with collinear FM order and another with c-AFM order. Therefore, the staggered nature of the DMI favors a noncollinear magnetic phase between SIO and SRO but collinear phases within the SIO and SRO layers themselves, which are more metallic compared to the nc-AFM order in pure SIO and AFM order in pure SRO. To understand the impact of magnetic-reconstruction on electronic structure, we compared the DOSs for three-layer systems with different structural configurations in their respective magnetic ground states: c-AFM SRO, nc-AFM SIO, and two types of SIO/SRO interfaces with c-AFM SIO and FM SRO.

**Fig. 4:**
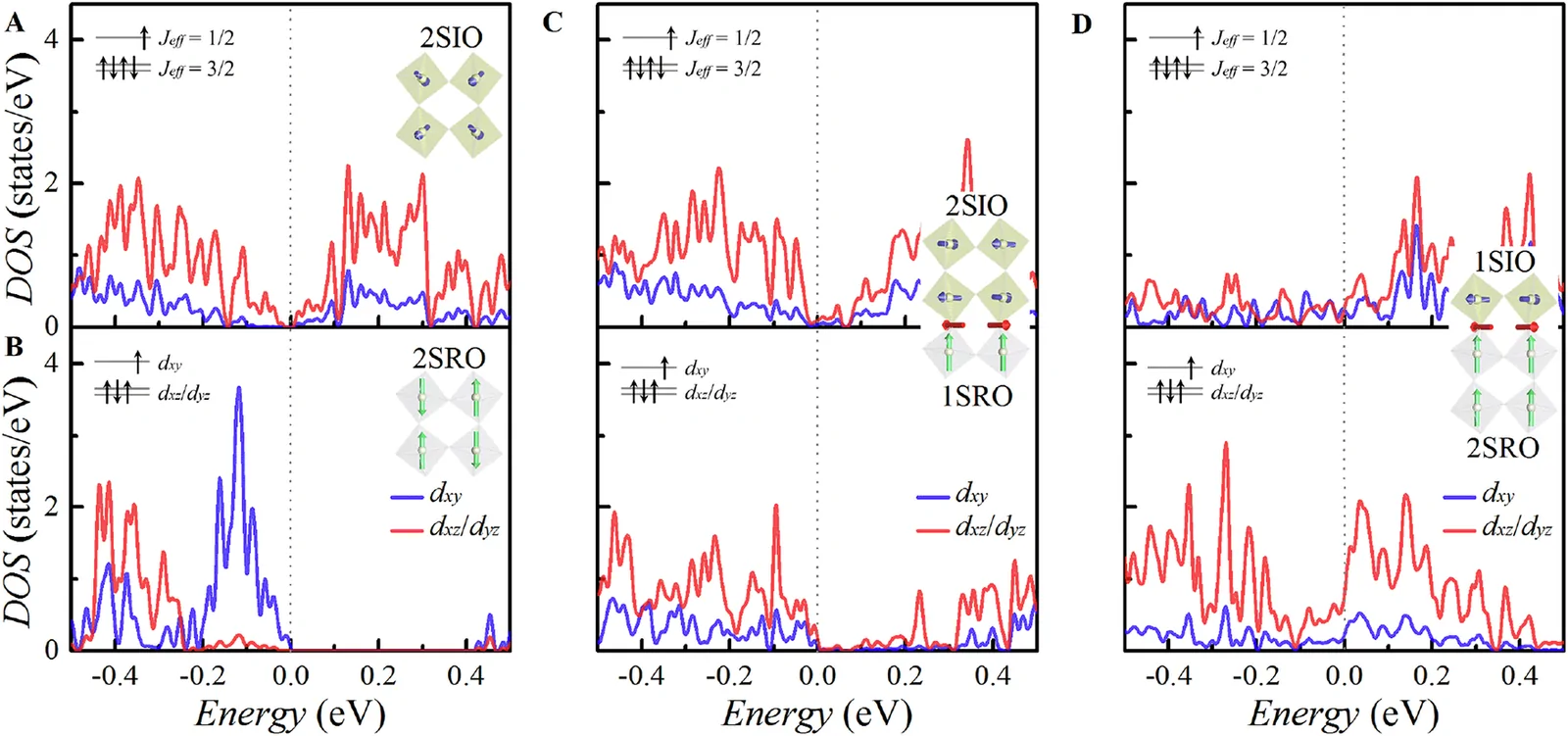
Orbital-resolved density of states (DOSs) of individual thin films and heterostructures. DOS comprised of *d*_*xy*_ (blue) and degenerate *d*_*xz*_*/d*_*yz*_ (red) orbitals are depicted for the films of (**A**) 2SIO and (**B**) 2SRO, and the heterostructures of (**C**) 2SIO/1SRO and (**D**) 1SIO/2SRO. Magnetic order and electronic configurations are indicated in the insets, where SIO and SRO crystal structures are drawn in dark yellow and gray-white. Spin ordering for Ir and Ru are represented by blue and green arrow, respectively. At the Ir-Ru interface, the staggered DMI (red arrow) stabilizes non-collinear spin orientation between Ir and Ru.

Orbital-resolved DOSs are presented in Fig. 4 for individual insulating 2-u.c. SIO and SRO films, and metallic three-layer heterostructures. A clear gap between the valence band and conduction band in 2SIO and 2SRO confirms the insulating nature at the critical thickness for the IMT. However, the pronounced spectral weight of the DOS at the Fermi level in the 2SIO/1SRO heterostructure indicates the emergence of a metallic state. The DOS calculations for the 1SIO/2SRO heterostructure exhibit a similar behavior to that of the 2SIO/1SRO system. Additionally, they reveal a DOS spectrum that continuously crosses the Fermi level, further substantiating the emergence of a metallic state. Moreover, the calculations indicate higher conductivity in the 1SIO/2SRO heterostructure compared to 2SIO/1SRO, which is consistent with our experimental observations.

## Discussion

Electronic reconstruction at the SIO/SRO interface has previously attracted considerable interest, particularly in the context of suppressing the dimensionality-driven metal-insulator transition (mit) in ultrathin SrIrO₃. Elemental intermixing can, in principle, occur in heterostructures composed of different oxide layers. Indeed, such effects may have a particularly pronounced impact in ultrathin heterostructures consisting of only 1–2 u.c. layers, where even limited interdiffusion could substantially modify the local electronic and magnetic structure. Although we cannot completely rule out the possibility of elemental interdiffusion at the SIO/SRO interface, we have minimized this possibility by carefully optimizing and lowering the growth temperature, combined with precise layer-by-layer growth control monitored by reflection high-energy electron diffraction (RHEED), as shown in Figs. S1 and S2. However, the metal-insulator transition (MIT) in SIO/SRO heterostructures has already been discussed in the literature. J. N. Nelson and co-workers attributed the emergence of metallicity to hybridization between Ir and Ru t_2__g_ states at the SIO/SRO interface, together with interfacial charge transfer, resulting in a metallic state even when a single SrIrO₃ layer was grown on metallic SrRuO₃^26^. However, the observed behavior cannot be satisfactorily explained by a simple charge-transfer-only mechanism. In contrast to what would be expected for significant interfacial charge transfer, our previous study of a closely related heterostructure^31^ revealed only very small shifts of the SRO-derived bands and no clear rigid-band displacement. This suggests that the emergent metallicity is governed by a more complex interfacial electronic reconstruction. Our work focuses on engineering magnetic interactions at the SIO/SRO interface as a route to emergent metallicity. Unlike previous studies, where metallic ferromagnetism in ultrathin SRO was achieved through optimization of the SRO layer itself^32–35^, both isolated ultrathin SRO and SIO layers in our case are insulating and show no ferromagnetic order. Metallicity and ferromagnetism emerge only after formation of the SIO/SRO heterostructure, pointing to an interfacial origin. The metallic state is also distinct from the conductivity observed in oxide interfaces such as LAO/STO^36–39^, where electronic reconstruction leads to the formation of a two-dimensional electron gas. While our results do not demonstrate a strict Slater-type transition, they are consistent with a reconstructed interfacial state governed by the interplay of Ru–Ir exchange coupling, magnetocrystalline anisotropy, and staggered Dzyaloshinskii–Moriya interactions. Together, these interactions provide a plausible microscopic framework for the coupled magnetic and electronic reconstruction at the SIO/SRO interface. In particular, the emergence of DMI in SIO/SRO heterostructures arises from strong spin-orbit coupling combined with broken inversion symmetry at the interface. Unlike conventional changes in magnetic order driven by exchange interactions influenced by strain or doping^40^, the staggered DMI originating from oxygen octahedral distortions plays the key role here. In our calculations, it converts the nc-AFM order in SIO into a c-AFM configuration and simultaneously drives an AFM-to-FM transition in SRO, consistent with the experimentally observed coexistence of emergent metallicity and ferromagnetic signatures, highlighting the importance of spin-lattice coupling in determining the interfacial magnetic ground state^41–43^. The DMI also induces a chiral spin component perpendicular to the preferred exchange and anisotropy directions, reflecting broken mirror and rotational symmetries at the interface (Supplementary Sections III-IV). Similar chiral spin structures have been observed in ultrathin SrRuO₃ films^44^, suggesting a shared microscopic origin. The competition among DMI, magnetocrystalline anisotropy, and anisotropic Ru–Ir exchange ultimately stabilizes the emergent ferromagnetic metallic state in the SIO/SRO heterostructures. Since it has been shown that the DMI is tunable through external electric and magnetic fields^45^, we speculate that the SIO/SRO system could serve as a platform for dynamic control of chiral magnetic textures, opening opportunities for chiral spintronics^46,47^ and novel electronic functionalities^48^.

In summary, our work shows that ultrathin SIO/SRO heterostructures exhibit emergent metallicity and ferromagnetic ordering, in contrast to the insulating and non-ferromagnetic behavior of the isolated ultrathin constituent layers. Together with theoretical calculations, these observations support a scenario in which staggered interfacial Dzyaloshinskii–Moriya interactions and the associated magnetic-reconstruction play an important role in the emergence of the metallic state. These findings identify interfacial magnetic interactions as a powerful tuning parameter for correlated electronic phases in spin–orbit-coupled oxide heterostructures, opening new opportunities for the rational design of quantum oxide materials with tailored electronic and magnetic functionalities for future spintronic and information-processing technologies.

## Methods

### Fabrication of heterostructures

SrIrO_3_/SrRuO_3_ (SIO/SRO) heterostructures were grown as bilayers, including 1u.c.-SIO/2u.c.-SRO (1SIO/2SRO), 2-u.c.-SIO/1-u.c.-SRO (2SIO/1SRO), and 2-u.c.-SIO/2-u.c.-SRO (2SIO/2SRO). All heterostructures were epitaxially grown on TiO_2_-terminated [001]-SrTiO_3_ (STO) substrates via pulsed laser deposition (PLD) assisted by reflection high-energy electron diffraction (RHEED) (See Figure S1, Supplementary Information). Polycrystalline SIO and SRO targets were ablated using a KrF excimer laser and Nd:YAG solid-state laser. SRO and SIO layers were successively grown at 650 °C in 0.15 mbar oxygen pressure. The laser fluence was 1.5 J/cm^2^ with a repetition rate of 2 Hz. Layer-by-layer growth was ensured by monitoring in-situ RHEED patterns and oscillations throughout deposition. Following deposition, heterostructures were annealed at 650 °C for 30 min in 1 mbar oxygen pressure to promote full oxidation and then cooled to room temperature at a cooling rate of 10 °C/min while maintaining oxygen pressure. For magnetic and electric transport measurements, a 15-u.c. STO capping layer was deposited on the grown ultrathin films prior to annealing to prevent degradation under ambient conditions.

### Magnetic and transport measurements

Magnetic measurements were performed ex-situ at 10 K using a Quantum Design superconducting quantum interference device (SQUID). Transport measurements were conducted using a physical property measurement system (PPMS) in a van der Pauw configuration (See Figure S2 and S3, in Supplementary Information).

### ARPES measurements

In-situ ARPES measurements were performed at the SIS beamline of the Swiss Light Source, Paul Scherrer Institute, utilizing a combined growth and spectroscopy setup. ARPES data were acquired at 10 K using circularly right-polarized synchrotron light with an energy of 82 eV under ultrahigh vacuum ( < 1 × 10^−10^ mbar). Low-energy electron diffraction (LEED) patterns were obtained after ARPES measurements. During the ARPES measurements, circularly polarized light was used to enhance the photoemission intensity and improve the signal-to-noise ratio in the ultrathin SIO/SRO heterostructures measured in-situ.

### First-principles calculations

Although precise structural and magnetic configurations cannot be fully resolved experimentally, the 2 × 2 periodicity observed in LEED measurements indicates the coexistence of oxygen octahedral rotations and tilts in the heterostructure. We therefore present the detailed distorted structural and magnetic phases as theory-guided interpretations that are consistent with the available structural evidence.

First-principles density functional theory (DFT) calculations were performed using the planewave VASP package^49,50^ within the local spin density approximation (LSDA) and employing the Perdew-Zunger parametrization^51^ of Ceperley-Alder data^52^ for the exchange-correlation functional. Core-valence electron interactions were described using the projector augmented wave (PAW) method^53^. On-site Coulomb interaction *U* and Hund’s exchange *J*_*H*_ were included for Ru, Ir, and Ti 3d orbitals within the LSDA + *U* approach, using a rotationally invariant scheme with *U* = 5 eV and *J*_*H*_ = 0.15 *U* (see Supplementary Information Sections Ⅱ to Ⅳ for details). To simulate experimental conditions, we modeled STO-SRO, STO-SIO, and STO-SRO-SIO heterostructures with SrO termination using a slab geometry with a 20 Å vacuum layer. The STO thickness was fixed at 3-unit cells (u.c.), while SRO-SIO layers were varied with 2 to 4 u.c.. A plane wave cutoff of 480 eV was used. Brillouin zone integration was performed using 8 × 8 × 1 and 12 × 12 × 1 *k*-point grids for geometric relaxation and density of states/total energy/charge/magnetization calculations, respectively. Spin-orbit coupling (SOC) was included in all calculations except for geometric relaxation, which was performed in the non-relativistic limit for computational efficiency^54^. Here, we study properties in the fully distorted phase with the in-plane lattice constant fixed to that of STO (3.905 Å). Internal degrees of freedom were optimized by minimizing total energy to below 3 × 10^−5 ^eV and Hellmann-Feynman forces to below 20 meV/Å. Illustrations of the magnetic configurations at the interfaces are presented in Supplementary Information, Figures S7–S11. The full set of calculations is presented in Supplementary Information, Figures S3–S6.

## Supplementary information

Transparent Peer Review file

Supplementary Information

## Data Availability

All data are available in the main text or the supplementary materials.
