# Supplementary material for: Interfacial magnetic reconstruction and emergent metallicity in oxide heterostructures
Source: Commun Mater. 2026 Jul 14;7(1):299. doi: 10.1038/s43246-026-01275-x (PMC13736518; doi:10.1038/s43246-026-01275-x)
Supplement: Supplementary file 1 [file 43246_2026_1275_MOESM1_ESM.pdf]

nature portfolio

Peer Review File

Interfacial Magnetic Reconstruction and Emergent Metallicity in Oxide Heterostructures

Corresponding Author: Professor Milan Radovic

This manuscript has been previously reviewed at another journal. This document only contains information relating to versions considered at Communications Materials.

This file contains all reviewer reports in order by version, followed by all author rebuttals in order by version.

Version 0:

Reviewer comments:

Reviewer #1

(Remarks to the Author)
The manuscript reports emergent metallicity and ferromagnetism in ultrathin $SrIrO_3/SrRuO_3$ heterostructures and attributes the effect primarily to interfacial DMI–driven magnetic reconstruction.
The work is promising and potentially suitable for Communications Materials, but major revision is required.
1. Interfacial intermixing in ultrathin PLD-grown superlattices
The heterostructures consist of 1–2 u.c. layers grown by PLD, where cation intermixing at the interface is essentially unavoidable. At this length scale, even a single intermixed layer would substantially modify the local electronic and magnetic structure.
The authors should explicitly discuss the likelihood of Ru–Ir intermixing at the interface and how this may affect the observed metallicity and ferromagnetism. In particular, intermixing could lead to local Ru-rich or Ir-diluted environments, which may independently promote itinerancy or ferromagnetism, complicating the interpretation in terms of a purely magnetic DMI-driven mechanism.
If available, STEM-EELS, STEM-EDX, or quantitative XPS depth profiling should be provided or discussed. If such data are not available, the authors should clearly state this limitation and discuss its implications. At minimum, the conclusions should be softened to acknowledge that interfacial chemical reconstruction may contribute alongside magnetic reconstruction.
2. Relation to prior work on ultrathin SRO films
There is a substantial body of work on ultrathin SRO, including studies by Changyoung Kim's group and others, showing thickness-driven metal–insulator transitions, magnetic reorientation, and emergent metallic states.
Several experimental signatures reported here (metallicity, ferromagnetism, ARPES spectral weight near Fermi level) appear qualitatively similar to those observed in ultrathin SRO films without SIO.
The manuscript should explicitly compare the present results with these prior studies and clarify: What features cannot be explained by known ultrathin SRO physics alone? Why the presence of SIO is essential, beyond acting as a structural or electronic proximity layer? Without a clear distinction, it remains ambiguous whether the observed behavior is genuinely interface-specific or simply a modified manifestation of known SRO thin-film physics.
3. Experimental evidence for the distorted phases invoked in theory
The theoretical analysis relies on specific distorted structural and magnetic phases, including changes in octahedral rotations and spin configurations.
It is not clear whether any of these predicted distortions are directly observed experimentally. Can signatures of the proposed distorted phases be identified in STEM (e.g., octahedral tilt patterns), LEED, or diffraction data? If direct confirmation is not feasible, the authors should frame the distorted phases more clearly as theoretical interpretations, and explicitly state the lack of direct experimental verification.
4. Role of charge transfer versus magnetic reconstruction
The authors emphasize that the metallicity is not driven by charge transfer, yet direct experimental evidence ruling this out is limited. Can core-level shifts (XPS) or layer-resolved DOS analysis provide stronger support? The discussion should more carefully distinguish magnetic-interaction–driven effects from electronic proximity or hybridization effects, especially given prior reports on SIO/SRO interfaces.

Reviewer #2

(Remarks to the Author)
The manuscript by Zengxing Lu et al., “Interfacial Magnetic Reconstruction and Emergent Metallicity in Oxide Heterostructures,” investigates changes in the metal–insulator transition and magnetic behavior in SIO/SRO heterostructures. The authors propose an intriguing scenario in which a magnetic interaction drives the metal–insulator transition. While the topic itself is interesting and timely, I believe that several experimental and theoretical aspects do not sufficiently support the central claims of the manuscript.

In particular, at the growth temperature of 650 °C, the system is expected to be in a clearly nonmagnetic state. Under these conditions, it is difficult to expect magnetic effects to play a decisive role until the temperature is lowered well below ~100 K, where magnetic signatures begin to emerge. This raises concerns about the causal link between magnetism and the observed electronic reconstruction.

My specific comments on the experimental details are as follows:

1) When the SRO thickness is reduced to 1–2 unit cells, is there clear experimental evidence that the system becomes an antiferromagnetic insulator? How can the authors experimentally distinguish between a nonmagnetic insulator and an antiferromagnetic insulator in this ultrathin limit?

2) Several previous studies have reported metallic behavior in monolayer or bilayer SRO systems. A discussion and citation of these works would be important for context, for example:

Appl. Phys. Lett. 127, 022402 (2025)

Phys. Rev. B 105, 054429 (2022)

Phys. Rev. Lett. 103, 057201 (2009)
The manuscript would benefit from a clearer comparison with these results and an explanation of the apparent discrepancies.

3) What is the motivation for using circularly polarized light in the ARPES measurements? This choice should be justified more explicitly, especially in relation to the claimed magnetic effects.

4) In the ARPES analysis, the interpretation of the relative weight of the coherent peak is not sufficiently quantitative. Is it possible to extract and compare this relative weight in a more quantitative manner? In the current version, the distinction is difficult to assess.

5) The direction of the magnetic field in the magnetization measurements is critically important, especially given the expected magnetic anisotropy in these systems. Additional measurements, such as Hall or magnetoresistance data, or magnetization measured along two orthogonal directions, would significantly strengthen the magnetic claims.

Overall, based on the current experimental evidence (setting aside the theoretical calculations, which are known to be highly sensitive to the choice of U and J parameters), there does not appear to be sufficient support to clearly distinguish between antiferromagnetic and ferromagnetic order. More importantly, convincing evidence that magnetism is the driving mechanism behind the metal–insulator transition is lacking. If the transition is magnetic in origin, evidence analogous to a Slater-type transition, where an electrical transition occurs concomitantly with magnetic ordering below a well-defined magnetic transition temperature, would be necessary.

For these reasons, I find it difficult to recommend publication in its current form. However, if the authors can provide substantially stronger experimental evidence addressing the points above, I would be open to reconsidering the manuscript in a revised version.

Version 1:

Reviewer comments:

Reviewer #1

(Remarks to the Author)
The authors have addressed most of my questions raised in the previous round of review. The manuscript reports emergent metallicity and ferromagnetism in ultrathin $SrIrO_3$/$SrRuO_3$ heterostructures and attributes these effects primarily to interfacial DMI-driven magnetic reconstruction. Although some of the claims are still not fully supported by direct evidence, the authors have made a reasonable effort to rule out alternative explanations. Therefore, I believe that the main conclusions of the manuscript are adequately supported by the available experimental data.

Reviewer #2

(Remarks to the Author)
The manuscript has been significantly improved and is close to being suitable for publication. However, before acceptance, the authors should cite the previous DFT studies reporting antiferromagnetic $SrRuO_3$ ((Phys. Rev. Lett. 124, 026401 (2020); Adv. Mater. 32, 1905815 (2019); Phys. Rev. B 79, 140407(R) (2009); Phys. Rev. B 107, 064418 (2023)) that were mentioned in their response to Comment 1. Including these references in the main text would provide important context for the discussion and strengthen the manuscript.

Open Access This Peer Review File is licensed under a Creative Commons Attribution 4.0 International License, which permits use, sharing, adaptation, distribution and reproduction in any medium or format, as long as you give appropriate credit to the original author(s) and the source, provide a link to the Creative Commons license, and indicate if changes were made.
In cases where reviewers are anonymous, credit should be given to 'Anonymous Referee' and the source.
The images or other third party material in this Peer Review File are included in the article's Creative Commons license, unless indicated otherwise in a credit line to the material. If material is not included in the article's Creative Commons license and your intended use is not permitted by statutory regulation or exceeds the permitted use, you will need to obtain permission directly from the copyright holder.
To view a copy of this license, visit https://creativecommons.org/licenses/by/4.0/

We would like to sincerely thank both Reviewers for their careful evaluation of our manuscript and for their constructive and insightful comments. We highly appreciate the time and effort invested in the review process. We have carefully considered all remarks and suggestions, and we believe that the revised version of the manuscript has been significantly strengthened as a result.

In the following, we provide a detailed point-by-point response to all comments and describe the corresponding modifications introduced in the revised manuscript. The manuscript has been thoroughly revised in accordance with the Reviewers' comments and the explanations provided in this response letter. All modifications made to the main text are highlighted in blue for clarity. Additional experimental data and supporting analyses have also been included in the revised Supplementary Information.

Reviewer #1

The manuscript reports emergent metallicity and ferromagnetism in ultrathin $SrIrO_3$/$SrRuO_3$ heterostructures and attributes the effect primarily to interfacial DMI-driven magnetic reconstruction. The work is promising and potentially suitable for Communications Materials, but major revision is required.

Authors: We thank the Reviewer for the positive evaluation of our work and for recognizing its potential significance and suitability for Communications Materials. We appreciate the constructive feedback and have carefully addressed all points raised by the Reviewer in the following text.

Comment 1*. Interfacial intermixing in ultrathin PLD-grown superlattices*

The heterostructures consist of 1-2 u.c. layers grown by PLD, where cation intermixing at the interface is essentially unavoidable. At this length scale, even a single intermixed layer would substantially modify the local electronic and magnetic structure. The authors should explicitly discuss the likelihood of Ru-Ir intermixing at the interface and how this may affect the observed metallicity and ferromagnetism. In particular, intermixing could lead to local Ru-rich or Ir-diluted environments, which may independently promote itinerancy or ferromagnetism, complicating the interpretation in terms of a purely magnetic DMI-driven mechanism. If available, STEM-EELS, STEM-EDX, or quantitative XPS depth profiling should be provided or discussed. If such data are not available, the authors should clearly state this limitation and discuss its implications. At minimum, the conclusions should be softened to acknowledge that interfacial chemical reconstruction may contribute alongside magnetic reconstruction

Answer: We appreciate the Reviewer's comment regarding possible Ru-Ir intermixing in these ultrathin bilayers. This is an important consideration for 1-2 u.c. oxide heterostructures. Our current data, however, argue against intermixing being the primary origin of the emergent metallicity and ferromagnetism. In particular, we systematically compared samples grown at different deposition temperatures and found that heterostructures grown at 650 °C reproducibly show metallic transport behavior, whereas samples 700 °C become significantly more insulating (see Figure A1 below).

Since stronger cation diffusion would be expected at higher growth temperatures, this trend suggests that enhanced intermixing is not favorable for the observed metallic state. In addition, the *in-situ* RHEED patterns display clear layer-by-layer oscillations throughout the growth, consistent with well-controlled epitaxy and optimized conditions.

While direct chemical profile such as STEM-EELS, STEM-EDX, or quantitative XPS depth profiling is not available for the present samples, the available evidence does not support interfacial chemical reconstruction as the dominant mechanism behind the observed behavior. We have clarified this point in the revised manuscript and now state more explicitly that a limited degree of intermixing cannot be completely exclude (**Page 7, Paragraph 3, Lines 3-8**).

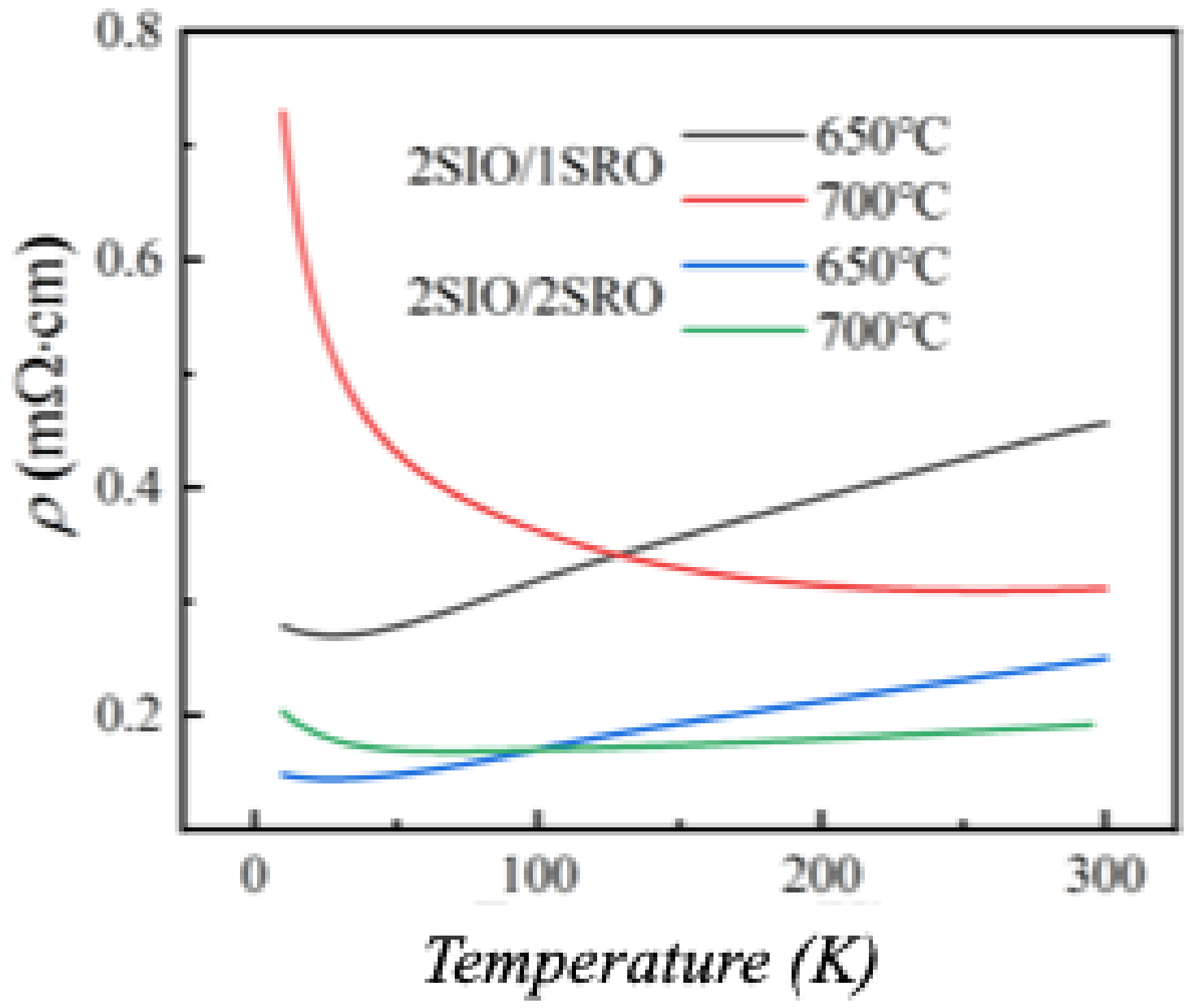

Figure A1 | Temperature-dependent resistivity of SIO/SRO heterostructures grown at different deposition temperatures. Compared with samples grown at 650 °C, heterostructures deposited at 700 °C exhibit substantially more insulating transport behavior.

Comment 2*. Relation to prior work on ultrathin SRO films*

There is a substantial body of work on ultrathin SRO, including studies by Changyoung Kim's group and others, showing thickness-driven metal-insulator transitions, magnetic reorientation, and emergent metallic states. Several experimental signatures reported here (metallicity, ferromagnetism, ARPES spectral weight near Fermi level) appear qualitatively similar to those observed in ultrathin SRO films without SIO. The manuscript should explicitly compare the present results with these prior studies and clarify: What features cannot be explained by known ultrathin SRO physics alone? Why

the presence of SIO is essential, beyond acting as a structural or electronic proximity layer? Without a clear distinction, it remains ambiguous whether the observed behavior is genuinely interface-specific or simply a modified manifestation of known SRO thin-film physics.

Answer: We thank the Reviewer's insightful comment on the relation between our results and previous studies of ultrathin SRO. We agree that metallicity, ferromagnetism, and finite spectral weight near E_F have been reported in ultrathin SRO films under carefully optimized conditions.

The key distinction in our work is that, under our specific growth and strain conditions, the isolated constituent layers do not show the same behavior as the heterostructure. In our measurements, both isolated ultrathin SRO and SIO remain insulating and show strongly suppressed spectral weight near the Fermi level, whereas the SIO/SRO bilayer exhibit clear metallic transport, finite spectra weight at E_F, and ferromagnetic hysteresis. In this sense, the emergent behavior in the bilayer cannot be accounted for by know ultrathin-SRO physics alone.

This also clarifies why the presence of SIO is essential in our system. SIO is not acting merely as a passive structural support or generic proximity layer; rather, it introduces strong spin-orbit coupling and contributes directly to the interfacial symmetry breaking and magnetic/electronic coupling at the SIO/SRO interface. Our first-principles calculations indicate that these interface-specific ingredients are associated with a reconstructed magnetic state and the appearance of metallicity in the heterostructure. We have revised the Discussion to make this distinction from prior ultrathin-SRO studies more explicit (**Page 8, Paragraph 2, Lines 2-8**).

Comment 3. *Experimental evidence for the distorted phases invoked in theory*

The theoretical analysis relies on specific distorted structural and magnetic phases, including changes in octahedral rotations and spin configurations. It is not clear whether any of these predicted distortions are directly observed experimentally. Can signatures of the proposed distorted phases be identified in STEM (e.g., octahedral tilt patterns), LEED, or diffraction data? If direct confirmation is not feasible, the authors should frame the distorted phases more clearly as theoretical interpretations, and explicitly state the lack of direct experimental verification.

Answer: We agree that the structural and magnetic phases used in the theoretical analysis should be connected experiment as clearly as possible. In the present work, the main experimental support of structural distortion comes from the LEED measurements. As shown in the Supplemental Fig. S1, the SIO/SRO bilayers exhibit a 2×2 surface periodicity, most clearly in the 2SIO/2SRO heterostructure and more weakly in 1SIO/2SRO and 2SIO/1SRO bilayers. This 2×2 pattern is consistent with the presence of octahedral rotation/tilt-related structural distortions of the type considered in our calculations. At the same time, we fully acknowledge that LEED does not uniquely determine the full octahedral rotation and tilt pattern, nor does it directly resolve the magnetic configuration. We therefore present the detailed distorted structural and magnetic phases as theory-guided interpretations that are consistent with the available

structural evidence, rather than as directly established experimental facts. This point is now stated more clearly in the **Method D** of the revised main text and **Supplementary Information**.

Comment 4. *Role of charge transfer versus magnetic reconstruction*
The authors emphasize that the metallicity is not driven by charge transfer, yet direct experimental evidence ruling this out is limited. Can core-level shifts (XPS) or layer-resolved DOS analysis provide stronger support? The discussion should more carefully distinguish magnetic-interaction-driven effects from electronic proximity or hybridization effects, especially given prior reports on SIO/SRO interfaces.

Answer: The Reviewer raise an important point concerning the distinction between charge transfer, interfacial hybridization, and magnetic reconstruction at the SIO/SRO interface. Our current data do not support substantial net charge transfer as the primary origin of the emergent metallicity.

First, the layer-resolved DOS analysis in Supplementary Fig. S6 shows that the finite DOS at the Fermi level appear together with the reconstructed interfacial magnetic/electronic state and is distributed across the heterostructure layers. This behavior is more naturally understood as an interfacial reconstruction effect than as a simple charge-transfer-only scenario.

Second, in our recent ARPES study of a closely related SIO/SRO heterostructure system [*Phys. Rev. Mater.* 9, 074403 (2025)], we tracked a representative SRO-derived band as a function of thickness and found only very small changes in its band position, without the clear rigid-band shift typically expected for substantial interfacial charge transfer (Figure A2).

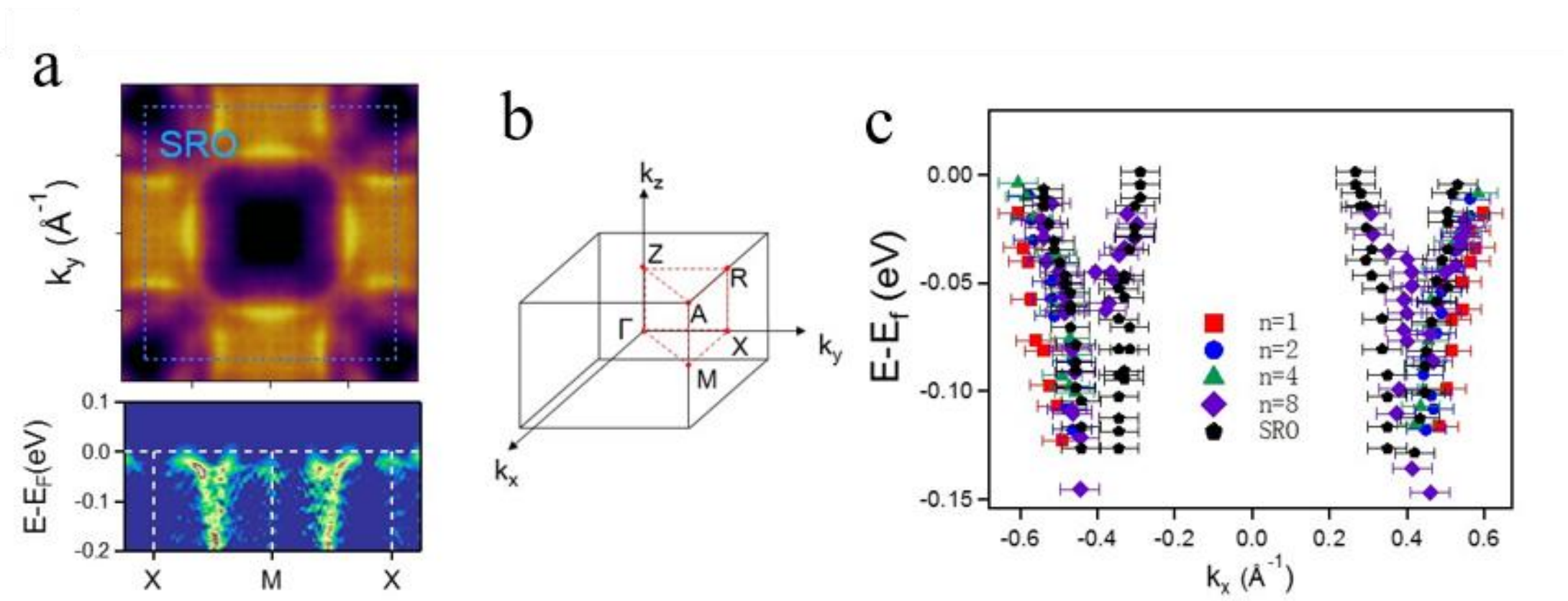

Figure A2 | Representative ARPES band tracking in a related SIO/SRO heterostructure system [Phys. Rev. Mater. 9, 074403 (2025)]. Extracted SRO-derived band positions for different SRO thicknesses show only weak variation and no obvious rigid-band shift, arguing against substantial net interfacial charge transfer.

This comparison does not exclude a limited amount of charge redistribution in the present ultrathin bilayers, but it argues against large net charge transfer as the dominant mechanism.

However, we do not rule out a contribution from interfacial hybridization or electronic proximity effects, especially in view of preview reports on SIO/SRO interfaces. We have therefore adjusted the wording throughout the revised manuscript to state this point more carefully: the observed behavior is not well explained by a simple charge-transfer-only mechanism and is more consistent with an interfacial reconstruction scenario in which magnetic coupling plays a central role, potentially together with hybridization effects (**Page 7, Paragraph 3, Lines 11-14**).

Reviewer #2:

The manuscript by Zengxing Lu et al., "Interfacial Magnetic Reconstruction and Emergent Metallicity in Oxide Heterostructures," investigates changes in the metal-insulator transition and magnetic behavior in SIO/SRO heterostructures. The authors propose an intriguing scenario in which a magnetic interaction drives the metal-insulator transition. While the topic itself is interesting and timely, I believe that several experimental and theoretical aspects do not sufficiently support the central claims of the manuscript.

In particular, at the growth temperature of 650 ℃, the system is expected to be in a clearly nonmagnetic state. Under these conditions, it is difficult to expect magnetic effects to play a decisive role until the temperature is lowered well below ~100 K, where magnetic signatures begin to emerge. This raises concerns about the causal link between magnetism and the observed electronic reconstruction.

Answer: We thank Reviewer #2 for the careful reading of our manuscript and for raising this important concern regarding the relationship between magnetism and the emergent metallic state in the SIO/SRO heterostructures. We agree that establishing the causal connection between magnetic reconstruction and the observed electronic properties is a central and nontrivial issue in this work.

We would like to clarify that our interpretation does not imply that magnetic order is already present during film growth at 650 °C. Rather, the growth temperature is relevant primary for establishing the structural quality and interface configuration of the heterostructure. The proposed magnetic mechanism concerns the low-temperature electronic and magnetic ground state of the fully growth bilayer, where the interfacial exchange interactions, spin-orbital coupling, and structural distortions can become important upon cooling.

However, we agree that the experimental evidence does not justify an overly strong claim of a purely magnetic-driven metal-insulator transition. We have therefore revised the manuscript to state this point more carefully. In the revised version, we describe the observed metallicity as being closely associated with, and consistent with interfacial magnetic reconstruction, rather than as definitive proof of a purely magnetic-driven MIT. This point is also revised in **Abstract** too.

We have addressed the Reviewer's specific concerns point-by-point below.

***Comment 1**. When the SRO thickness is reduced to 1-2 unit cells, is there clear experimental evidence that the system becomes an antiferromagnetic insulator? How can the authors experimentally distinguish between a nonmagnetic insulator and an antiferromagnetic insulator in this ultrathin limit?*

Answer: We thank the Reviewer for this important comment. We agree that, based on the experimental techniques used in the present study, we cannot unambiguously distinguish an antiferromagnetic insulating state from a nonmagnetic insulating state in isolated 1-2 u.c. SRO. In this ultrathin limit, a compensated AFM state would carry essentially no net moment, and its magnetic signal is expected to be too small to be resolved reliably by conventional magnetometry. A definitive distinction between these two cases would require more specialized probes, such as resonant magnetic X-ray

scattering or X-ray magnetic linear dichroism, which were not available in the present work.

We have therefore revised the manuscript to avoid overstating this point. In the revised version, we describe isolated ultrathin SRO more carefully as an insulating state that is consistent with an AFM ground state suggested by previous studies and supported by our calculations, rather than as an experimentally established AFM phase (**Page 4, Paragraph 2, Lines 6-10; Page 5, Paragraph 3, Lines 7-12**).

This interpretation is based on three considerations. First, our transport and ARPES measurements directly show that isolated 1-2 u.c. SRO is insulating, with strongly suppressed spectral weight near the Fermi level. Second, previous experimental and theoretical studies have reported AFM or AFM-tending ground states in ultrathin SRO (*Phys. Rev. Lett.* 124, 026401 (2020); *Adv. Mater.* 32, 1905815 (2019); *Phys. Rev. B* 79, 140407(R) (2009); *Phys. Rev. B* 107, 064418 (2023)). Third, our first-principles calculations identify an AFM insulating configuration as the energetically stable ground state among the magnetic configurations considered for isolated ultrathin SRO.

Importantly, the central experimental observation of this work does not rely solely on a direct proof of AFM order in isolated SRO. Rather, therefore, it is the clear contrast between the isolated ultrathin constituent layers, which remain insulating and do not exhibit ferromagnetic ordering (hysteresis is absent), and the SIO/SRO bilayer, which shows emergent metallic transport together with ferromagnetic signatures. We have clarified this point in the revised manuscript (**Page 8, Paragraph 2, Lines 2-8; Page 8, Paragraph 3, Lines 1-8**).

Comment 2. *Several previous studies have reported metallic behavior in monolayer or bilayer SRO systems. A discussion and citation of these works would be important for context, for example: Appl. Phys. Lett. 127, 022402 (2025); Phys. Rev. B 105, 054429 (2022); Phys. Rev. Lett. 103, 057201 (2009). The manuscript would benefit from a clearer comparison with these results and an explanation of the apparent discrepancies.*

Answer: We thank the Reviewer for pointing out these important earlier studies. We agree that the previous reports of metallicity in monolayer and bilayer SRO films need to be discussed more explicitly in order to place out results in the proper context. In the revised manuscript, we have now added the suggested references and expanded the discussion accordingly (**Page 8, Paragraph 2**).

We would like to emphasize that these earlier studies do not contradict our observations but rather highlight how sensitively the electronic ground state of ultrathin SRO depends on sample conditions such as substrate choice, epitaxial strain, capping, stoichiometry, and disorder. In several of the cited works, metallicity in ultrathin SRO was stabilized by using lattice-matched substrates, carefully optimized growth conditions, or capping layers that help preserve the itinerant ferromagnetic state of SRO down to the ultrathin limit.

By contrast, in our samples under the specific growth (and strain) conduction used here, isolated 1-2 u.c. SRO is insulating, as directly shown by both transport and ARPES measurements. The same is true for isolated ultrathin SIO. The key observation is therefore not simply that a thin SRO-containing structure can become metallic, but that

both individual constituent layers are insulating, whereas the SIO/SRO bilayer becomes metallic and develops ferromagnetic hysteresis. This contrast cannot be explained by the intrinsic behavior of isolated ultrathin SRO under our experimental conditions alone. This is also why the SIO layer is essential in our system. The SIO layer is not acting merely as a passive cap or structural support (a buffer layer); rather, it provides strong spin-orbital coupling and enables interface-specific electronic and magnetic reconstruction. Our calculations further suggest that interfacial magnetic reconstruction is an important ingredient in the emergence of the metallic state, although we now describe this point more cautiously in the revised manuscript. In particular, we no longer present the data as evidence for a universally or purely magnetic-driven transition in ultrathin SRO, but instead as support for an interface-specific reconstruction scenario in the SIO/SRO bilayer.

Comment 3*. What is the motivation for using circularly polarized light in the ARPES measurements? This choice should be justified more explicitly, especially in relation to the claimed magnetic effects.*

Answer: We thank the Reviewer for this useful question. We agree that the motivation for using circularly polarized light in the ARPES measurements should explained more clearly. The primary reason for choosing circularly polarized light was experimental sensitivity. Because the samples studied here are ultrathin (1-2 u.c.), the photoemission signal is intrinsically weak, and obtaining sufficient spectral weight near the Fermi level is challenging. Under our measurement conditions, circular polarization provided stronger photoemission intensity and better matrix-element contrast for the low-energy states of interest, which improved the overall signal-to-ratio of the in situ ARPES data. In addition, for a spin-orbital-coupled interface such as SIO/SRO, circularly polarized light can be advantageous for accessing Ru- and Ir-derived near -E_F states and possible interfacial hybridized features. However, we would like to emphasize that in the present work the use of circular polarization was not intended as a direct probe of magnetic order, and we do not base our magnetic conclusion on any circular-dichroism analysis. Rather, the ARPES measurements are used here to compare the presence of absence of spectral weight near the Fermi level in isolated ultrathin layers and in the bilayers heterostructure. We have clarified this point in the revised manuscript (**Page 6, Paragraph 1, Lines 5-10**).

Comment 4*. In the ARPES analysis, the interpretation of the relative weight of the coherent peak is not sufficiently quantitative. Is it possible to extract and compare this relative weight in a more quantitative manner? In the current version, the distinction is difficult to assess.*

Answer: We thank the Reviewer for this helpful suggestion. We agree that the original presentation of the ARPES data was too qualitative in discussing the relative coherent spectral weight near the Fermi level.

In the revised manuscript, we have therefore added a more quantitative analysis of the X-point EDCs shown Fig, 3A.iii-C.iii. Using the same fitting procedure for all three heterostructures, we extracted the integrated spectral weights of the coherent and

incoherent components and defined a coherent spectral fraction, $f_{\text{coh}} = \frac{W_{\text{coh}}}{W_{\text{coh}}+W_{\text{incoh}}} \times 100\%$. The extracted f_{coh} increases systematically from 26% (2SIO/1SRO) to 28% (1SIO/2SRO) and 55% (2SIO/2SRO), making the trend in low-energy coherence more explicit.

We have revised the corresponding discussion in the main text and figure caption accordingly (**Page 6, Paragraph 1, Lines 15-21**).

Comment 5. *The direction of the magnetic field in the magnetization measurements is critically important, especially given the expected magnetic anisotropy in these systems. Additional measurements, such as Hall or magnetoresistance data, or magnetization measured along two orthogonal directions, would significantly strengthen the magnetic claims.*

Answer: We thank the Reviewer for this helpful suggestion. We agree that the field orientation should be stated explicitly when discussing the magnetization of these ultrathin heterostructure. In the revised manuscript, we now clarify that the *M-H* hysteresis loops shown in Fig. 2B were measured with the magnetic field applied perpendicular to the film plane.

To further strengthen the magnetic analysis, we have added out-of-plane anomalous Hall effect (AHE) measurements for all three heterostructures. Clear AHE hysteresis loops are observed in each case. Since the AHE is directly sensitive to the out-of-plane component of the magnetization, these measurements provide independent evidence for ferromagnetism with a substantial perpendicular magnetic component in the bilayers (Fig. A3b). The AHE results are consistent with the square-like out-of-plane *M-H* loops shown in Fig. 2B.

In addition, we performed field-direction-dependent magnetoresistance (MR) measurements for a representative sample 2SIO/1SIO (Fig. A3c). The larger MR response under out-of-plane field compared with in-plane field is consistent with predominantly out-of-plane magnetic anisotropy.

Given the ultrathin thickness of the samples, direct magnetization measurements along two orthogonal directions are experimentally challenging. We therefore rely on the combined *M-H*, AHE, and directional MR results to support the anisotropy discussion. These new data have been added to Fig. S3, and the corresponding discussion has been expanded in the revised manuscript (**Page 5, Paragraph 3, Lines 12-15**) and **Supporting Information**.

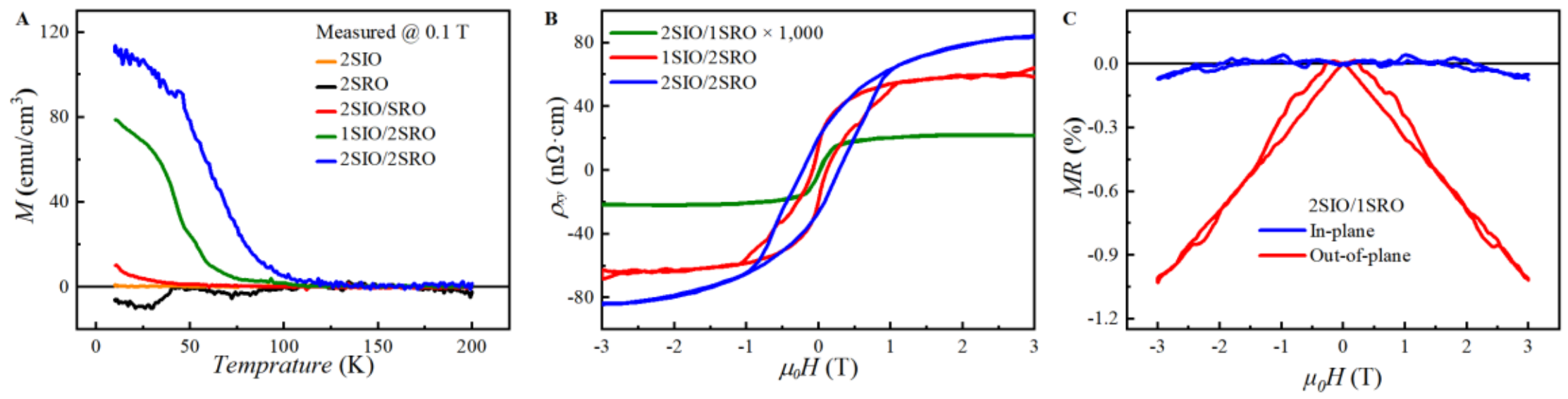

Figure A3 | Additional magnetic characterization of the SIO/SRO heterostructures. (a) Temperature-dependent magnetization M(T) measured at 0.1 T. (b) Out-of-plane anomalous Hall resistivity measured at 10 K. (c) Magnetoresistance of the 2SIO/1SRO heterostructure measured at 10 K with the magnetic field applied in plane and out of plane.

Comment 6*: Overall, based on the current experimental evidence (setting aside the theoretical calculations, which are known to be highly sensitive to the choice of U and J parameters), there does not appear to be sufficient support to clearly distinguish between antiferromagnetic and ferromagnetic order. More importantly, convincing evidence that magnetism is the driving mechanism behind the metal-insulator transition is lacking. If the transition is magnetic in origin, evidence analogous to a Slater-type transition, where an electrical transition occurs concomitantly with magnetic ordering below a well-defined magnetic transition temperature, would be necessary.*

Answer: We thank the Reviewer for this important summary comment. We agree that the experimental evidence presented in the original submission was not sufficient to establish a textbook Slater-type transition, nor to prove unambiguously that magnetism alone is the sole driving force behind the emergent metallic state. We have therefore revised the manuscript to present this point more carefully (**Page 8, Paragraph 2**).

We agree that the present data do not by themselves demonstrate a strict Slater-type transition with a sharply defined and concomitant magnetic and transport anomaly. Rather, they indicate a close correlation between the emergence of metallicity and the reconstructed magnetic state of the bilayer. For this reason, we have softened the wording throughout the revised manuscript: instead of claiming that magnetism is conclusively proven to “drive” the metal–insulator transition, we now state that the observed emergent metallicity is closely associated with, and consistent with, interfacial magnetic reconstruction.

We also agree that first-principles calculations, including the choice of interaction parameters, should not be taken as stand-alone proof of the mechanism. Accordingly, in the revised manuscript the calculations are presented as a theory-guided interpretation that provides a plausible microscopic framework for the coupled electronic and magnetic reconstruction at the SIO/SRO interface. In addition, we now explicitly acknowledge that interfacial hybridization/electronic proximity effects, and possibly a limited degree of chemical reconstruction, may also contribute to the observed behavior.

Taken together, we believe that the revised manuscript now more accurately reflects

both the strength and the limitations of the present evidence. Our main conclusion is therefore framed more cautiously: the emergent metallic state in ultrathin SIO/SRO heterostructures is not well explained by conventional ultrathin-SRO physics or simple charge transfer alone and is most consistently understood as an interface-driven reconstructed state in which magnetic coupling plays an important role.

For these reasons, I find it difficult to recommend publication in its current form. However, if the authors can provide substantially stronger experimental evidence addressing the points above, I would be open to reconsidering the manuscript in a revised version.

Answer: We thank the Reviewer for this constructive and encouraging final remark. In response to the concerns raised, we have substantially revised the manuscript by adding new anomalous Hall effect and anisotropic magnetoresistance measurements, expanding the discussion of magnetic anisotropy and interfacial coupling, and clarifying the respective roles of magnetic reconstruction, hybridization, and possible chemical reconstruction. We have also softened several statements throughout the manuscript to more clearly distinguish experimentally established observations from theory-guided interpretations.

We hope that our responses and additional results adequately address the Reviewer's concerns and that the revised manuscript will be suitable for reconsideration.
