# Supplementary material for: Interfacial magnetic reconstruction and emergent metallicity in oxide heterostructures
Source: Commun Mater. 2026 Jul 14;7(1):299. doi: 10.1038/s43246-026-01275-x (PMC13736518; doi:10.1038/s43246-026-01275-x)
Supplement: Supplementary file 2 [file 43246_2026_1275_MOESM2_ESM.docx]

**Supplementary Information**

**Interfacial Magnetic Reconstruction and Emergent Metallicity in Oxide Heterostructures**

Zengxing Lu, Jiatai Feng, Xuan Zheng, You-Guo Shi, Run-Wei Li, Nicholas C. Plumb, Carmine Autieri, Mario Cuoco, Zhiming Wang, and Milan Radovic

**Address correspondence to:**

[zhiming.wang@nimte.ac.cn](mailto:zhiming.wang@nimte.ac.cn)

[autieri@magtop.ifpan.edu.pl](mailto:autieri@magtop.ifpan.edu.pl)

[mario.cuoco@spin.cnr.it](mailto:mario.cuoco@spin.cnr.it)

[milan.radovic@psi.ch](mailto:milan.radovic@psi.ch)

**This file includes:**

Section Ⅰ. Sample fabrication and characterization

Figs. S1 and S2

Section Ⅱ. *Ab-initio* analysis of the SIO-SRO-STO ultrathin films

Section Ⅲ. Symmetries of the spin Hamiltonian

Section Ⅳ. Numerical evaluation of the Hamiltonian parameters

Figs. S3 to S11

Section Ⅰ. Sample fabrication and characterization

Figure S1A shows the Reflection High-energy Electron Diffraction (RHEED) data of a series of SrRuO_3_-SrIrO_3_ (SRO-SIO) heterostructures on [001]-SrTiO_3_ (STO) substrates, namely 2SIO/1SRO, 1SIO/2SRO, and 2SIO/2SRO, where 1 (2) represents 1 (2) unit cell (u.c.), respectively. Through the observation of RHEED oscillations during growth, it can be inferred that these films are grown layer-by-layer at the atomic level. The surface structure of the films is further confirmed by Low Energy Electron Diffraction (LEED), as shown in Fig. S1B-1D. The clear 2×2 surface periodicity is identified in 2SIO/2SRO bilayer, pointing towards the presence of out-of-plane tilting and in-plane rotation. In 2SIO/1SRO and 1SIO/2SRO bilayers, the 2×2 periodicities were faint but observed, indicating that the out-of-plane tilting in 2SIO/1SRO and 1SIO/2SRO still exist, albeit only were partially suppression compared to that in the 2SRO/2SIO. This 2×2 pattern indicates the presence of octahedral rotation- and tilt-related structural distortions of the type considered in the following theoretical calculations.

**Fig. S1. Growth of the SIO-SRO bilayer films. (A)** RHEED oscillations during depositing 2SIO/2SRO, 1SIO/2SRO and 2SIO/1SRO films. LEED patterns of **(B)** 2SIO/2SRO, **(C)** 1SIO/2SRO and (D) 2SIO/1SRO films. The patterns are captured under high vacuum after growth. The octahedral out-of-plane tilting and in-plane rotation produce 2 × 2 surface periodicity, which has been observed in all of the bilayer films.

The growth condition was optimized by systematically comparing the metallic transport properties of the samples grown at different deposition temperatures. We find that heterostructures grown at 650 °C reproducibly show metallic transport behavior, whereas samples 700 °C become significantly more insulating (seen in Figure S2). Thus, all subsequent films are prepared at 650 ℃.

**Fig. S2 Temperature-dependent resistivity of SIO/SRO heterostructures grown at different deposition temperatures.** Compared with samples grown at 650 °C, heterostructures deposited at 700 °C exhibit substantially more insulating transport behavior.

As shown in Figure S3, the ferromagnetism of the SIO/SRO bilayer films are identified via measuring temperature-dependent magnetization (*M-T*) and out-of-plane anomalous Hall effect (AHE) measurements. The bilayer samples exhibit strongly enhanced ferromagnetic behavior with the magnetic response increasing systematically with SRO thickness, while the ultrathin 2SIO and 2SRO do not show magnetic signals, confirming the antiferromagnetic-to-ferromagnetic transition (See Fig. S3A). The AHE measurements presented in Fig. S3b show clear hysteresis loops for all samples, providing additional evidence for ferromagnetic ordering. Importantly, the saturated anomalous Hall resistivity (*ρ_AHE_*) increases, systematically from 2SIO/1SRO (0.029 nΩ·cm) to 1SIO/2SRO (64 nΩ·cm) and 2SIO/2SRO (84 nΩ·cm). This evolution closely follows the enhancement of both the ferromagnetic moment and metallic conductivity observed in the *M-H*, and *ρ-T* measurements (Figure 2A and 2B). Furthermore, the pronounced squareness of both the out-of-plane AHE hysteresis loops strongly indicates that the heterostructures are dominated by perpendicular magnetic anisotropy (PMA).

To further investigate the anisotropic magnetic behavior, we additionally performed both in-plane and out-of-plane magnetoresistance (MR) measurements for 2SIO/1SRO heterostructure. This particular sample was selected because it lies close to the critical thickness regime associated with the predicted magnetic and electronic phase transition, including the theoretically proposed spin reorientation from in-plane AFM to out-of-plane FM ordering in the SRO layer. The MR results (Fig. S3C) reveal a pronounced anisotropy: the out-of-plane MR reaches approximately 1% at 3 T, which is significantly larger than the in-plane MR (0.065% at 3 T). This strong directional dependence provides further transport evidence for dominant PMA in the heterostructures.

**Fig. S3. Magnetic transport properties of SRO, SIO and SIO-SRO films.** (**A**) Temperature-dependent electric transition (*M-T*) measured at 0.1T after magnetized at 7 T completely. (**B**) Out-of-plane anomalous Hall effect (AHE), and (**C**) magnetoresistance (MR) effect along different directions.

Taken together, the combined *M-H*, *M-T*, AHE, and anisotropic MR measurements consistently support the conclusion that all investigated SIO/SRO heterostructures exhibit perpendicular magnetic anisotropy.

**Section Ⅱ. *Ab-initio* analysis of the SIO-SRO-STO ultrathin films**

In this section, we report the *ab-initio* study of the SIO-SRO-STO heterostructure by considering the electronic properties and the derivation of the magnetic model for the spin of the Ir and Ru atoms.

A. Pure phases

The SRO bulk is itinerant ferromagnetic (FM), but shows a bandwidth-driven metal-insulator transition (MIT). The system is antiferromagnetic insulator (AFMI) for 2 u.c. or less while it is FM metallic (FMM) for 3 u.c. or more, as illtrustrated in Figs. 1C and S4A. The 2-u.c. FM SRO phase is metallic (Fig. S4B) but reducing the thickness to 1 u.c., the FM SRO phase becomes insulating. However, this was not observed so far since the 1 u.c. insulating FM phase is energetically unstable. When we add the spin-orbit coupling (SOC), the critical value of *U* is shifted, indeed, the SOC favors the AFM insulating phase since tends to produce more splittings. This metal-insulator transition is reproduced for *U_Ru_* ⩾ 3.36 eV. The energy difference between the FM and AFM phase at *U* = 3.36 is 0.06 meV per formula unit, while the bandgap of the AFM phase is 0.41 eV.

The SIO bulk is a topological semimetal with the metallicity protected by the combination of time-reversal symmetry and crystalline symmetry. When we go in the 2D limit, the system becomes magnetic due to the band reduction and this gives the chance to open the gap. The magnetic order in SIO is AFM with canting, while it is impossible to stabilize the FM order in pure SIO compounds. More in detail, the SIO presents two competing antiferromagnetic phases with slightly different electronic and magnetic properties. We have an insulating AFM magnetic phase with strong canting of the spin and a more metallic AFM phase without canting of the spins, as shown in Fig. S5. Without the spin canting the magnetic component *S_y_* and *S_z_* are zero, but the *S_x_* component does not increase resulting in a reduced magnetic moment in the AFM phase without canting. Therefore, the reduction of the magnetic moment produces the disappearance of the gap. Also in this system, there is a bandwidth-driven metal-insulator transition. The system is AFM insulator for 3 u.c. or less while it is semimetallic for 4 u.c. or more. Within our numerical setup, the metal-insulator transition is reproduced for *U_Ir_* ⩾1.31 eV, quantitative differences with previously reported results are attributed to the use of a different exchange-correlation functional. [1,2] The energy difference between the two magnetic phases of SIO is 0.97 meV at *U* = 1.31 eV, while the band gap of the non-collinear antiferromagnetic (nc-AFM) phase is 7 meV. These so accurate values of *U* are necessary for the MIT that is reproducible for values of *U* in a very narrow range of *U*, however, the magnetic phase is much more stable.

In Fig. 1, we have shown how the magnetic phases of the homogeneous thin films. The magnetic ground state of the SIO is non-collinear antiferromagnetic (nc-AFM), while the ground of the SRO is collinear antiferromagnetic (c-AFM) within DFT. When we join the SRO and SIO compounds creating the interface, the interface Dzyaloshinskii-Moriya interaction (DMI) changes the magnetic phases of both sides of the bilayer. The SIO becomes c-AFM while the SRO becomes collinear FM (c-FM). In both cases, the metallic phase is favored, as illustrated in Fig. S6. This changes the critical thickness of the metal-insulator transition, and we observe the insulating phase just for the 2 u.c. case 1SIO/1SRO (Fig. S7).

B. Effect of the DMI

The DMI between Ru and Ir tends to push towards an angle of 90 degrees between Ru and Ir. This configuration is favored by the different local magnetocrystalline anisotropy of Ru and Ir, and the instability of SRO and SIO towards different magnetic orders. This interplay makes the DMI very effective with an angle of 90 degree between Ru and Ir spin comments. Considering the staggered order, the increase of the metallicity produced by the DMI is two-fold. From one side of the heterostructure, the DMI favors a c-AFM phase in the SIO which is more metallic. On the other side of the heterostructure, the DMI pushes the SRO in the FM phase which is more metallic. The DMI between a magnetic atom and a magnetic heavy atom is rarely seen. Usually, the DMI is between light magnetic atoms via a heavy metal. In have the magnetic configuration of the heterostructures in insets and the corresponding DOSs.

There are also other effects (less exotic than the DMI) that favor the metallicity. The gap of the SRO is opened by the electronic correlation, while the proximity with the SIO reduces the *U*/*W* due to the larger hopping Ru-Ir [3]. The gap of the SIO is opened by the SOC, while the proximity with the SRO reduced the effective SOC. The metallicity is higher in the inner layers or interface layers, so we expect the metallicity to come from them. The less metallic is 1SIO/1SRO, then 2SIO/1SRO and then 1SIO/2SRO is the most metallic, therefore SRO is the major source of metallicity. Once we stabilize the metallicity, the Coulomb repulsion is screened both in SRO and SIO making the value of the Coulomb repulsion of the order of 1 eV for both compounds like in bulk or thick films [4,5].

C. Low values of *U_Ir_*

The magnetization on the Ir has two sources: one intrinsic due to the onsite *U_Ir_* and one due to the magnetic proximity of the SRO. When the *U_Ir_* is below 1.2 eV, the magnetic proximity dominates and the Ru spins along the *z*-direction induce a small ferromagnetic component along *S_z_* on the same fashion as the SrRuO_3_/Sr_2_RuO_4_ interface.[6] When *U_Ir_* is 1.2 eV or above, the intrinsic source of the magnetism dominates and we have the in-plane magnetic moment with AFM order. On the other side, if we increase the *U_Ir_*, we increase the moment of the Ru and consequently the proximity effect. Therefore, the AFM insulating phase can be observed only in the ultrathin limit where the reduction of the electronic bandwidth increases the effective value of the local Coulomb repulsion *U_Ir_* on the iridium atoms

D. 2 unit cells of pure and mixed phases

We report the DOS of the pure phases of SRO and SIO. We can observe as they are insulating. The bandgap of the AFM phase of SIO is 7 meV at *U_Ir_* = 1.31 eV. The DMI effect is not strong enough to create metallicity also in 1SIO/1SRO, therefore we have an insulator with net magnetic moment, as shown in Fig. S7.

**Section Ⅲ. Symmetries of the spin Hamiltonian**

The space group 62 (to which both SIO and SRO belong) has mirror symmetry and rotation symmetry. The mirror symmetry is broken by the interface, while the rotation symmetry is broken by the spontaneous symmetry breaking of the DMI that selects a given chirality. Therefore, no symmetries are left for the SIO/SRO system.

We calculate the magnetic properties of the simpler case 1SIO/1SRO that is insulating, we expect that the metallicity rising in the 3 u.c. cases will change these properties but not dramatically.

We consider just the Hamiltonian terms relevant for this configuration excluding the interlayer DMI and the spin along *y*. We also avoid to calculate the magnetic coupling between the Ir atoms which is always AFM. We name the Ir atoms as atom 1 and 2, while the Ru atoms are atom 3 and 4 as in Fig. S8A. We split the spin Hamiltonian into the intraplane and interplane terms, the intraplane Hamiltonian is further split in symmetric and antisymmetric respect to the exchange of atoms. Moreover, the symmetric term is split into the isotropic and anisotropic. Within these considerations the total spin Hamiltonian reads:

$H=H^{inter}+H_{SYM}^{intra, iso}+H_{SYM}^{intra, aniso}+H_{ASYM}^{intra}$	(1)

The interplane Hamiltonian in the supercell is

$H^{inter}=4J_{34}\vec{S}_{3}\cdot\vec{S}_{4}+K^{Ir}\left( {cos}^{2}\left( \theta_{1} \right)+{cos}^{2}\left( \theta_{2} \right) \right)+K^{Ru}\left( {cos}^{2}\left( \theta_{3} \right)+{cos}^{2}\left( \theta_{4} \right) \right)+E_{0}$	(2)

where *θ_i_* is the azimuthal angle respect to the *z*-axis of the *i^th^* atom.

In the case of isotropic interaction, the intraplane diagonal (diag) terms would be:

$H_{SYM}^{intra,diag}=J_{13}^{iso}\left( \vec{S}_{1}\cdot\vec{S}_{3} \right)+J_{24}^{iso}\left( \vec{S}_{2}\cdot\vec{S}_{4} \right)=J_{13}^{iso}(\vec{S}_{1}\cdot\vec{S}_{3}+\vec{S}_{2}\cdot\vec{S}_{4})$	(3)

however, in this system the diagonal (diag) symmetric coupling is not isotropic, therefore we have:

$H_{SYM}^{intra,diag}=J_{13x}^{diag}\left( S_{1x}S_{3x}+S_{2x}S_{4x} \right)+J_{13z}^{diag}(S_{1z}S_{3z}+S_{2z}S_{4z})$	(4)

Since it is impossible to describe the magnetic properties just with the DMI, we need to add the non-diagonal (nd) symmetric coupling that is:

$$H_{SYM}^{intra,nd}=J_{13}^{nd}S_{1x}S_{3z}+J_{24}^{nd}S_{2x}S_{4z}+J_{13}^{nd}S_{1z}S_{3x}+J_{24}^{nd}S_{2z}S_{4x}$$

$=J_{13}^{nd}[\left( S_{1x}S_{3z}-S_{2x}S_{4z} \right)+\left( S_{1z}S_{3x}-S_{2z}S_{4x} \right)]$	(5)

while the antisymmetric magnetic coupling is:

$$H_{ASYM}^{intra}=\vec{D}_{13}\cdot\left( \vec{S}_{1}\times\vec{S}_{3} \right)+\vec{D}_{24}\cdot\left( \vec{S}_{2}\times\vec{S}_{4} \right)=\vec{D}_{13}\cdot(\vec{S}_{1}\times\vec{S}_{3}-\vec{S}_{2}\times\vec{S}_{4})$$

$=D[\left( S_{1x}S_{3z}-S_{2x}S_{4z} \right)-\left( S_{1z}S_{3x}-S_{2z}S_{4x} \right)]$	(6)

where we have used the properties that $J_{24}^{nd}$=-$J_{13}^{nd}$ and $\vec{D}_{24}$=-$\vec{D}_{13}$ due to the symmetry of the system. The DMI and $J^{nd}$ have a staggered symmetry. In the bulk perovskite, the DMI along the *c*-axis is $\vec{D}_{c}=(0,+D,0)$ presenting just the *y*-component that is orthogonal to the M-O-M plane in our reference system. For the SRO/SIO interface, the DMI consists of all three components but we can approximate the DMI as $\vec{D}_{13}\approx(0,+D,0)$.

The combination of the staggered DMI and $J^{nd}$, combined with the magnetic crystalline anisotropy (MCA), favors the ferromagnetic phase in the SRO.

**Section Ⅳ. Numerical evaluation of the Hamiltonian parameters**

We will calculate the energy of several magnetic configurations: 4 collinear and 5 non-collinear magnetic phases. We will have 8 parameters to determine: $J_{34}$, $K^{Ru}$, $K^{Ir}$, $E_{0}$, $J_{13x}^{diag}$ , $J_{13z}^{diag}$, $J_{13}^{nd}$ and D. The 5 non-collinear magnetic configurations are nc1, nc2, nc3, nc4 and nc5 shown in Figs. S8, S9 and S10. Starting from the configuration nc1, we can get nc2 inverting the Ir spins. Starting from the configuration nc1, we can get nc3 exchanging the Ir and Ru spins first-neighbor. Starting from the configuration nc1, we can get nc4 flipping the spin of one Ru atom. The two collinear magnetic configurations are c_x_FM, c_x_AFM, c_z_FM and c_z_AFM are represented in Figs. S11.

Summing equations (5) and (6) we have:

$H_{SYM}^{intra,nd}+H_{ASYM}^{intra}=\left( J_{13}^{nd}+D \right)\left( \hat{S}_{1x}\hat{S}_{3z}-\hat{S}_{2x}\hat{S}_{4z} \right)+\left( J_{13}^{nd}-D \right)\left( \hat{S}_{1z}\hat{S}_{3x}-\hat{S}_{2z}\hat{S}_{4x} \right)$	(7)

in the case of AFM Ir and FM Ru magnetic configuration, we have that one of the spins terms is zero, while the other is ±2. If the phase is collinear or both magnetic phases are AFM, the previous term is zero.

Assuming to map on a spin 1 Hamiltonian, the energies of the non-collinear magnetic configurations are the following:

$E_{NC1}=4J_{34}+2K^{Ru}+E_{0}+2(J_{13}^{nd}-D)$	(8)

If we exchange both Ir atoms, we change the sign to the coefficient of $J_{13}^{aniso}$ and D

$E_{NC2}=4J_{34}+2K^{Ru}+E_{0}-2(J_{13}^{nd}-D)$	(9)

If we exchange the spin of Ru and Ir, the antisymmetric part changes sign while the symmetric is not.

$E_{NC3}=4J_{34}+2K^{Ir}+E_{0}+2(J_{13}^{nd}+D)$	(10)

$E_{NC4}=4J_{34}+2K^{Ir}+E_{0}-2(J_{13}^{nd}+D)$	(11)

$E_{NC5}=-4J_{34}+2K^{Ru}+E_{0}$	(12)

To calculate $J_{13x}^{diag}$and $J_{13z}^{diag}$, we need to calculate the energy from collinear magnetic configurations along *x*(C_x_) and along *z*(C_z_), we choose the in-plane antiferromagnetic configurations that contains a ferromagnetic (FM) and antiferromagnetic (AFM) coupling between Ir and Ru as reported in Figs. S11. The equations for the collinear configurations are:

$E_{CzFM}=-4J_{34}+2J_{13z}^{diag}+2K^{Ir}+2K^{Ru}+E_{0}$	(13)

$E_{CzAFM}=-4J_{34}-2J_{13z}^{diag}+2K^{Ir}+2K^{Ru}+E_{0}$	(14)

$E_{CxFM}=-4J_{34}+2J_{13x}^{diag}+E_{0}$	(15)

$E_{CxAFM}=-4J_{34}-2J_{13x}^{diag}+E_{0}$	(16)

Finally, we have 8 linearly independent equations from which we can extract the microscopic parameters.

c1 is the ground state for low values of *U*, while the ground state becomes nc1 for high values of *U*.

The final ground state for 1-u.c. SIO is composed by the non-collinear ground state but with an Ir-component antiparallel to the Ru.

We report our results for *U* = 3.36 eV and *U* = 1.31 eV, but the following relations are obtained for close values of *U*:

$J_{13z}^{diag}\gg-J_{34}>-J_{13x}^{diag}>0$	(17)

$J_{13z}^{nd}>D>0$	(18)

Due to these results, the ground state is the configuration nc2 (or something similar). The value of DMI oscillating as a function of the number of layers as previously report by A. Hajr and coworkers [7].

The Ru-Ir magnetic coupling is antiferromagnetic if the spins are along *z*, while the Ru-Ir is ferromagnetic if spins are along *x*. We have a strongly anisotropic Heisenberg model that is barely observed in real materials. The antiferromagnetic phase with spin along *z* does not become the ground state because is not favored by the magnetocrystalline anisotropy of the Ir (*K_Ir_*) and the other non-collinear terms such as DMI. Basically, there is a competition between the Ru-Ir magnetic coupling vs. *K_Ir_*+DMI_(_*_Ru,Ir_*_)_. We plot the layer-resolved DOS for the ground state nc2 which is the ground state for the 1SIO/1SRO.

We have different MCA between the two compounds; this produces non-collinear order and strong DMI due to the 90 degrees angle. We have a very robust magnetic configuration with FM coupling between Ru spin with orientation along *z* and AFM coupling between Ir spin with orientation along *x*. The DMI favors the more metallic phases. Additionally, the electronic proximity helps metallicity. We have a competition between different magnetic effects like DMI and several magnetic exchanges. The structural part is not the dominant effect of the MIT in this heterostructure. Since the single layer of FM SRO and c-AFM SIO are insulating, the *K_Ir_* is insulating creating a unique two-dimensional insulating ferrimagnetic phase in oxide.

The non-collinear magnetic ground state has a vector chirality $\vec{S}_{Ir}\times\vec{S}_{Ru}$, as shown in Fig. S12, but the magnetic configuration is co-planar so has zero scalar spin chirality. The chirality of the ground state seems to be dependent on several factors (value of *U* and number of layers like the sign of the *D* vector in the Co/heavy-metal surface). The experiments show an exchange bias and a hysteresis loop with two coercive fields. Usually, these two coercive fields can be produced by non-collinear magnetic configuration.

**Fig. S4.** Layer-resolved density of state (DOS) of the antiferromagnetic **(A)** and ferromagnetic **(B)** phase of 2-u.c. SRO on STO at *U* = 3.36 eV. The spins are along *z* for both phases. These two competing phases are AFM insulating and FM metallic for 2 u.c., but they are both insulating for 1 u.c.

**Fig. S5.** Layer-resolved DOS of the **(A)** non-collinear antiferromagnetic (nc-AFM) and **(B)** collinear antiferromagnetic (c-AFM) phase of 3-u.c. SIO at *U* = 1.31 eV. The main component of the spins is along *x* for the non-collinear phase and the only component is along *x* for the collinear phase. These two competing phases are both insulating for 1 u.c.

**Fig.S6.** Layer-resolved DOS for **(A)** 2SIO/1SRO and **(B)** 1SIO/2SRO for the reported values of *U*. Both DOSs are metallic, the magnetic configurations are FM*_z_*/AFM*_x_* for both heterostructures. The Ir moments converged to zero in the case of 2 layers of SRO, this makes the 1SIO/2SRO system extremely metallic.

**Fig. S7**. **(A)** DOS of the FM*_z_*/AFM*_x_* phase of 1SIO/1SRO at the critical values of U. **(B)** DOS of the AFM*_z_* phase of 2SRO. **(C)** DOS of the AFM*_x_* phase of the 2SIO.

**Fig. S8.** Non-collinear magnetic configuration **(A)** number 1 and **(B)** number 2. In the first case, the spins of the Ir atoms point towards the same orientation of the tilting displacement of the apical oxygen, while in the second case they have the opposite orientation. The green and the blue arrows represent the Ru and Ir spins, respectively. The *y*-component of the DMI vector is represented in red.

**Fig. S9.** Non-collinear magnetic configuration **(A)** number 3 and **(B)** number 4. The green and the blue arrows represent the Ru and Ir spins, respectively. The *y*-component of the DMI vector is represented in red.

**Fig. S10.** Non-collinear configuration number 5 where the Ru is AFM. The green and the blue arrows represent the Ru and Ir spins, respectively. The *y*-component of the DMI vector is represented in red.

**Fig. S11.** Collinear magnetic configuration with **(A)** interlayer AFM coupling and **(B)** intralayer FM coupling. The green and the blue arrows represent the Ru and Ir spins, respectively. The *y*-component of the DMI vector is represented in red.

**Fig. S12.** The spin of the Ir points in the direction of the tilting of apical oxygen atom between Ru and Ir for the case of the 1SRO/1SIO, while goes in the opposite direction for the 1SRO/2SIO. For the case 2SRO/1SIO, the spin of the Ir atoms is zero.

**Reference:**

[1] D. J. Groenendijk, C. Autieri, J. Girovsky, M. C. Martinez-Velarte, N. Manca, G. Mattoni, A. M. R. V. L. Monteiro, N. Gauquelin, J. Verbeeck, A. F. Otte, M. Gabay, S. Picozzi, and A. D. Caviglia, Spin-orbit semimetal SrIrO_3_ in the two-dimensional limit, *Phys. Rev. Lett.* **119**, 256403 (2017).
[2] T. C. van Thiel, J. Fowlie, C. Autieri, N. Manca, M. Siˇ skins, D. Afanasiev, S. Gariglio, and A. D. Caviglia, Coupling lattice instabilities across the interface in ultrathin oxide heterostructures, *ACS Mater. Lett.* **2**, 389 (2020).
[3] D. J. Groenendijk, C. Autieri, T. C. van Thiel, W. Brzezicki, J. R. Hortensius, D. Afanasiev, N. Gauquelin, P. Barone, K. H. W. van den Bos, S. van Aert, J. Verbeeck, A. Filippetti, S. Picozzi, M. Cuoco, and A. D. Caviglia, Berry phase engineering at oxide interfaces, *Phys. Rev. Research* **2**, 023404 (2020).
[4] N. Manca, D. J. Groenendijk, I. Pallecchi, C. Autieri, L. M. K. Tang, F. Telesio, G. Mattoni, A.McCollam, S. Picozzi, and A. D. Caviglia, Balanced electron-hole transport in spin-orbit semimetal SrIrO_3_ heterostructures, *Phys. Rev. B* **97**, 081105 (2018).
[5] S. Roy, C. Autieri, B. Sanyal, and T. Banerjee, Interface control of electronic transport across the magnetic phase transition in SrRuO_3_/SrTiO_3_ heterointerface, *Sci. Rep.* **5**, 15747 (2015).
[6] O. Maistrenko, C. Autieri, G. Livanas, P. Gentile, A. Romano, C. Noce, D. Manske, and M. Cuoco, Inverse proximity effects at spin-triplet superconductor-ferromagnet interface, *Phys. Rev. Research* **3**, 033008 (2021).
[7] A. Hajr, A. Hariri, G. Manchon, S. Ghosh, and A. Manchon, Semirealistic tight-binding model for Dzyaloshinskii-Moriya interaction, *Phys. Rev. B* **102**, 224427 (2020).
